# scmFormer Integrates Large‐Scale Single‐Cell Proteomics and Transcriptomics Data by Multi‐Task Transformer

**DOI:** 10.1002/advs.202307835

**Published:** 2024-03-14

**Authors:** Jing Xu, De‐Shuang Huang, Xiujun Zhang

**Affiliations:** ^1^ Key Laboratory of Plant Germplasm Enhancement and Specialty Agriculture Wuhan Botanical Garden Chinese Academy of Sciences Wuhan 430074 China; ^2^ University of Chinese Academy of Sciences Beijing 100049 China; ^3^ Eastern Institute for Advanced Study Eastern Institute of Technology Ningbo 315200 China; ^4^ Center of Economic Botany Core Botanical Gardens Chinese Academy of Sciences Wuhan 430074 China

**Keywords:** multi‐task leaning, single‐cell data generation, single‐cell data integration, single‐cell multi‐omics, single‐cell protein, spatial multi‐omics, transformer

## Abstract

Transformer‐based models have revolutionized single cell RNA‐seq (scRNA‐seq) data analysis. However, their applicability is challenged by the complexity and scale of single‐cell multi‐omics data. Here a novel single‐cell multi‐modal/multi‐task transformer (scmFormer) is proposed to fill up the existing blank of integrating single‐cell proteomics with other omics data. Through systematic benchmarking, it is demonstrated that scmFormer excels in integrating large‐scale single‐cell multimodal data and heterogeneous multi‐batch paired multi‐omics data, while preserving shared information across batchs and distinct biological information. scmFormer achieves 54.5% higher average F1 score compared to the second method in transferring cell‐type labels from single‐cell transcriptomics to proteomics data. Using COVID‐19 datasets, it is presented that scmFormer successfully integrates over 1.48 million cells on a personal computer. Moreover, it is also proved that scmFormer performs better than existing methods on generating the unmeasured modality and is well‐suited for spatial multi‐omic data. Thus, scmFormer is a powerful and comprehensive tool for analyzing single‐cell multi‐omics data.

## Graphical abstract

scmFormer, a Transformer‐based model, employs multi‐task learning for single‐cell multi‐omics integration and unmeasured data generation. It excels in preserving shared information across diverse datasets, achieving a 54.5% higher average F1 score in cell‐type label transfer. Impressively scalable, scmFormer seamlessly integrates millions of cells on personal computers, outperforming existing methods in generating unmeasured modalities and excelling in spatial multi‐omic data analysis.

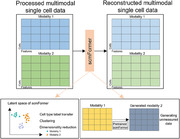

## 1 Introduction

Over the past few years, a great number of single‐cell multi‐omics sequencing technologies have surfaced, exhibiting the capacity to measure a range of cellular characteristics such as the transcriptome, epigenome, and proteome in individual cells simultaneously.^[^ ^1^, ^2^, ^3^, ^4^, ^5^, ^6^, ^7^, ^8^ ^]^ These advancements have facilitated thorough investigations into the heterogeneity of cells across multiple omics layers, bestowing comprehensive insights and promoting more precise classification of cell types and states.^[^ ^9^, ^10^, ^11^ ^]^

Proteins are central to all cellular processes and functions, playing a crucial role in shaping cell structure and enabling biochemical reactions as enzymes.^[^ ^12^, ^13^, ^14^ ^]^ Hence, in order to simultaneously capture single‐cell omics (transcriptome or epigenomics) and proteomics, some technologies have been developed and extensively applied in the analysis of immune systems.^[^ ^15^, ^16^, ^17^, ^18^ ^]^ However, the integration of such data is primarily challenged by three factors. First, there is a stronger presence of technical bias between gene expression and protein expression data compared to that between gene expression and chromatin accessibility data. Second, there is a significant disparity in the feature dimensions between these two modalities. Third, the overlap of shared features between these two modalities is limited, posing a further obstacle for some methods to integration or resulting in the inability of certain methods to merge them.

Recently, there has been a surge in the methods specifically developed for the analysis of multimodal single cell data.^[^ ^12^, ^19^, ^20^ ^]^ These methods can be categorized into three main groups: linear matrix decomposition methods employing canonical correlation analysis (Seurat^[^ ^9^ ^]^ BindSC^[^ ^21^ ^]^) or matrix factorization (Liger,^[^ ^22^, ^23^ ^]^ online iNMF^[^ ^24^ ^]^), manifold alignment methods (Pamona^[^ ^25^ ^]^ UnionCom^[^ ^26^ ^]^), and neural network methods (GLUE^[^ ^27^ ^]^ scJoint^[^ ^28^ ^]^ uniPort^[^ ^29^ ^]^ scVI^[^ ^30^ ^]^ totalVI^[^ ^31^ ^]^ sciPENN^[^ ^18^ ^]^ BABEL^[^ ^32^ ^]^ scMM^[^ ^33^ ^]^).

The majority of these models mainly focus on the integration of scRNA‐seq and single cell ATAC‐seq (scATAC‐seq) data while overlooking the incorporation of protein data. Furthermore, when confronted with large‐scale single cell multimodal data, each of these three categories of methods exhibits its own respective limitations. Linear matrix decomposition‐based methods can handle large‐scale datasets, with notable efficiency seen in approaches like Liger and online iNMF, but these methods often struggle to achieve satisfactory performance. Methods relying on manifold alignment are constrained to analyzing small‐scale datasets because of the inherent computational complexity they possess. Neural network‐based methods have gained popularity in analyzing large‐scale single‐cell data.^[^ ^34^, ^35^, ^36^ ^]^ These approaches primarily depend on generative models such as autoencoders^[^ ^37^ ^]^ variational autoencoders^[^ ^38^ ^]^ and generative adversarial networks^[^ ^39^ ^]^ Nevertheless, these methods demand significant computational resources in comparison to other types of methods. Additionally, the majority of these models are constrained to the analysis of bi‐omics data solely as a result of their architectural design. Moreover, a prominent characteristic of deep learning‐based methods resides in their difficulty in handling small‐scale datasets.

**Figure 1:**
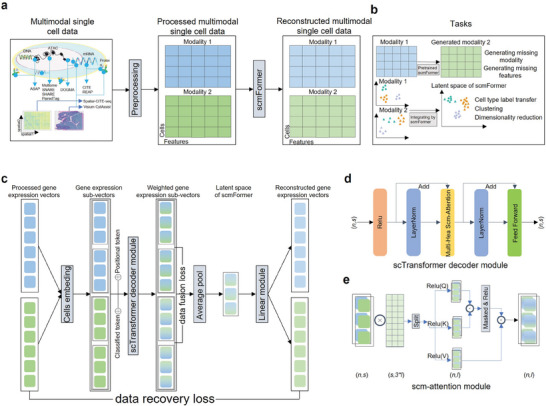
Overview of scmFormer. a) Broad schematic of scmFormer workflow. scmFormer demonstrates its capability to accommodate diverse single‐cell omics data as input. scmFormer accepts input in the form of expression vectors from different omics modalities and produces reconstructed expression profiles specific to each modality. Furthermore, scmFormer generates a joint representation encompassing all modalities for each cell. b) scmFormer possesses the capacity to integrate multimodal single‐cell data, thereby acquiring the latent representation for each modality. The latent representations are used to downstream tasks. scmFormer can also be employed to generate unmeasured modalities based on the available measured modalities. c) Structure of scmFormer. scmFormer is constructed upon the transformer, comprising three key modules: Cells Embedding, scTransformer Decoder, and Linear Layer. d) Within the scTransformer Decoder layer, the input consists of *n* sub‐vectors, each having dimensions of (*n, s*), and the resulting output also comprises *n* sub‐vectors with dimensions of (*n, s*). The scm‐attention network operates in parallel *H* times, enabling comprehensive information exchange. e) Within the scm‐attention Network, the input comprises *n* sub‐vectors, characterized by dimensions of (*n, s*), while the resulting output consists of *n* sub‐vectors with dimensions of (*n, l*). To obtain the query (*Q*), key (*K*), and value (*V*) matrices, linear projection is applied to these sub‐vectors.

The emergence of transformer‐based methods, incorporating attention mechanisms, has revolutionized scRNA‐seq analysis.^[^ ^36^, ^40^, ^41^, ^42^, ^43^ ^]^ Leveraging the robust GPT‐3.5 architecture, ChatGPT has demonstrated exceptional proficiency in natural language processing and dialogue systems^[^ ^44^ ^]^ Inspired by this impressive model and to address these concerns, we developed a single‐cell multimodal/multi‐task transformer approach called scmFormer for integrating single‐cell multimodal data and and proteomics data (**Figure** **1**a). In contrast to resource‐intensive transformer‐based models such as Geneform^[^ ^45^ ^]^ and scBert^[^ ^46^ ^]^ scmFormer overcomes the inherent limitations of transformer‐based^[^ ^47^ ^]^ models by adopting patches (sub‐vectors)^[^ ^36^ ^]^ and scm‐attention (Experimental Section). Additionally, we have devised a novel attention mechanism specifically for analyzing single‐cell multimodal data, named scm‐attention (Figure 1e and Experimental Section). scmFormer effectively addresses the complexities inherent in the integration of single‐cell proteomics and transcriptomics (epigenomics) by employing a dual‐task framework: 1) a data recovery task, which endeavors to maximize the similarity between the model's output data and the input data, thereby providing the well‐representation of cells; and 2) a data fusion task that facilitates the amalgamation of heterogeneous data modalities to extract shared information while preserving the modality‐specific information.

Through rigorous benchmarking and comprehensive analyses, we have demonstrated the accuracy, reliability, and scalability of scmFormer for single‐cell multimodal data. To the best of our knowledge, scmFormer represents a groundbreaking advancement by introducing transformer to analyze multimodal single‐cell data. The versatility of scmFormer allows its application to diverse multi‐omics single‐cell data, including transcriptoics‐plus‐epigenomics,^[^ ^6^, ^7^, ^8^ ^]^ and spatial transcriptomics‐plus‐proteomics datasets,^[^ ^48^, ^49^ ^]^ among others (Figure 1a).

## 2 Results

### 2.1 Multimodal Single Cell Data Analysis with scmFormer

scmFormer addresses the task of integrating single‐cell proteomics with other omics data, a significant challenge for many existing methods. Besides, scmFormer overcomes the problem of integrating large‐scale and complex single‐cell multimodal data, allowing for the derivation of latent representations specific to each modality. These latent representations are subsequently employed in downstream analysis tasks (Figure 1b). Furthermore, scmFormer enables the generation of unmeasured modality based on the measured one.

scmFormer is built upon the transformer, incorporating three key modules including Cells Embedding, scTransformer Decoder Layer, and Linear Layer (Figure 1c and Experimental Section). The objective of the Cells Embedding is to split gene (peak, protein) expression vectors into sub‐vectors suitable for utilization as input within the scTransformer Decoder. Furthermore, we incorporate encoded positional and categorical information into the input data. The scTransformer Decoder constitutes the central module of scmFormer, exhibiting the capacity to process sub‐vectors through scm‐attention mechanisms and feed‐forward neural networks (Figure 1d,e and Methods). The scTransformer Decoder generates latent representations specific to each modality. Subsequently, the Linear Layer is employed to receive the output of the scTransformer Decoder and facilitate the reconstruction of gene (peak, protein) expression.

In contrast to the domains of Natural Language Processing (NLP) and Computer Vision (CV), the field of biological data exhibits an elevated level of complexity. Accordingly, in each subsequent benchmarking experiment, we meticulously collect multiple datasets to rigorously evaluate the robustness and scalability of scmFormer.

### 2.2 scmFormer Excels in Single‐Cell Omics Plus Proteomics Datasets

Single‐cell proteomics data greatly contributes to the investigation of tumor heterogeneity and treatment resistance^[^ ^13^ ^]^ The integration of proteomic data with other omics datasets facilitates a substantial reduction in the workload for researchers, particularly in tasks such as cell type annotation within proteomics. Furthermore, the integration of multi‐batch single‐cell multi‐omics data significantly enhances data utilization efficiency. Additionally, integrating large‐scale datasets is currently an imperative for researchers. To evaluate the performance of scmFormer in the context of integrating single‐cell omics and proteomics datasets, we have designed four distinct benchmarking scenarios.

#### 2.2.1 Benchmarking on Single‐Cell Omics Plus Proteomics Datasets

**Figure 2:**
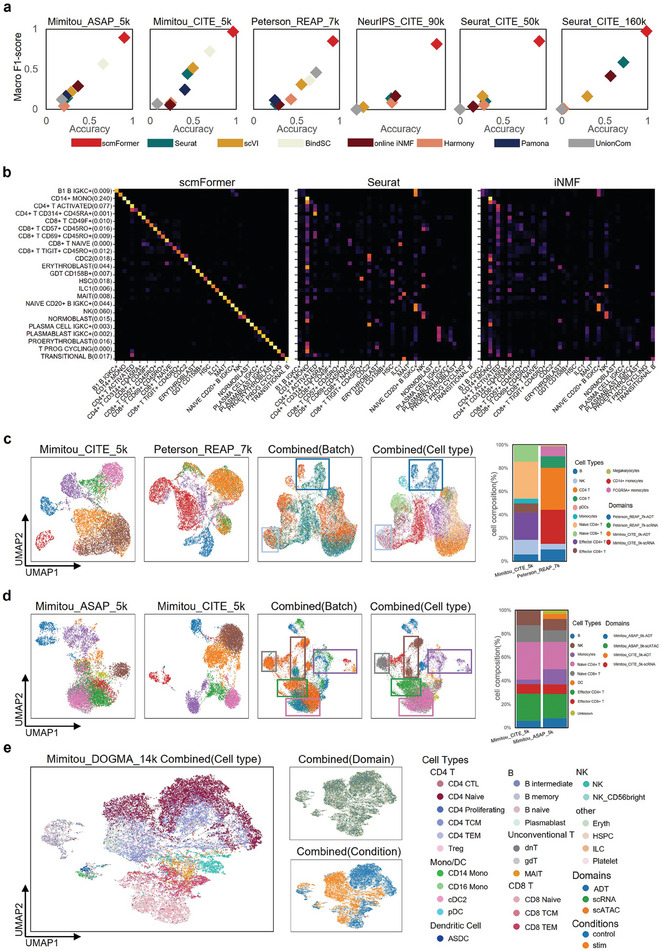
scmFormer enables effective analysis of single‐cell omics plus proteomics. a) Performance of methods measured by macro F1‐scores and accuracy. b) Confusion matrix heatmaps for results of scmFormer, Seurat, and GLUE on the theNeurIPS_CITE_90k. c) UMAP visualizations of the latent space for all cells of Mimitou_CITE_5k (first column) and Peterson_REAP_7k (second column), colored by cell types. Integrated latent space visualizations, colored by omics layers(third column) and cell types(fourth column). d) UMAP visualizations of the latent space for all cells of Mimitou_ASAP_5k (first column) and Mimitou_CITE_5k (second column), colored by cell types. Integrated latent space visualizations, colored by omics layers(third column) and cell types(fourth column). e) UMAP visualizations of the integrated latent space for all cells of Mimitou_DOGMA_14k, colored by cell types(left), omics layers(top right), experiment condition (bottom right).

We initially compared scmFormer with seven methods (Seurat,^[^ ^9^ ^]^ Harmony,^[^ ^50^ ^]^ Online iNMF,^[^ ^24^ ^]^ BindSC,^[^ ^21^ ^]^ scVI,^[^ ^30^ ^]^ Pamona,^[^ ^25^ ^]^ UnionCom^[^ ^26^ ^]^) across six datasets (Mimitou_ASAP_5k,^[^ ^5^ ^]^ Mimitou_CITE_5k,^[^ ^5^ ^]^ Peterson_REAP_7k,^[^ ^51^ ^]^NeurIPS_CITE_90k,^[^ ^34^ ^]^Seurat_CITE_50k,^[^ ^9^ ^]^ Seurat_CITE_160k^[^ ^9^ ^]^). As mentioned above, there are limitations in the number of proteins that can be simultaneously profiled. Therefore, it is difficult to annotate cell types of proteome data, regardless of the utilization of marker genes‐based methods or well‐annotated scRNA‐seq dataset‐based methods.^[^ ^52^, ^53^ ^]^ Hence, we proceeded to compare different methods on the task of cell‐type label transferring. Based on the experimental results depicted in **Figure** **2**a, it is evident that scmFormer exhibits superior performance compared to the other methods in terms of macro F1 score and accuracy across all datasets. On average, scmFormer exhibited a remarkable enhancement of 54.5% in the term of macro F1 score and 61.7% in the term of accuracy compared to the second‐best method. Furthermore, in the term of single‐cell level alignment, scmFormer outperforms the other methods by a significant margin (Figure S1a, Supporting Information). Additionally, the running time of scmFormer is satisfactory on a standard laptop (Figure S1b, Supporting Information). Figure 2b clearly demonstrates that scmFormer exhibits a substantial performance advantage over other methods on the large‐scale NeurIPS_CITE_90k dataset (Figure S2, Supporting Information). We also observed that the second‐best method, BindSC, is unable to handle large‐scale datasets (NeurIPS_CITE_90k, Seurat_CITE_50k, and Seurat_CITE_160k). To rigorously assess the performance of scmFormer, we extended scmFormer to consider scenarios wherein the distribution of cell counts across modalities is imbalanced (Supplementary Method S1, Supporting Information). To this end, we generated six distinct datasets characterized by varying degrees of imbalance from the NeurIPS_CITE_90k and Seurat_CITE_160k. These datasets served as benchmarking datasets. Despite the inherent challenges posed by uneven data, scmFormer demonstrated commendable adaptability, consistently outperforming competing methodologies across most datasets. Quantitatively, scmFormer achieved an average improvement of 12.2% in the macro F1 score and 14.6% in accuracy relative to the next best performing method (Figure S1c,d, Supporting Information). However, it is noteworthy that there remains substantial room for enhancement in the model's performance. These comparative analyses unequivocally demonstrate the remarkable performance of scmFormer in the integration of single‐cell omics with proteomics datasets (Figures S2–S7, Supporting Information).

#### 2.2.2 Integrating Multi‐Batch Paired Multi‐Omics Data

To overcome the high costs of multi‐omics sequencing technologies and achieve a comprehensive characterization of cellular and individual heterogeneity, integration of multi‐batch paired multi‐omics data is employed. First, we integrated two distinct datasets, Mimitou_CITE_5k and Peterson_REAP_7k, derived from human PBMCs, using different sequencing technologies. Additionally, these two datasets exhibit dissimilar feature dimensions, with a particularly limited overlap of only 36 common proteins. These pose challenges for integrating the two datasets, but scmFormer flawlessly integrates two paired datasets. As illustrated in Figure 2c, it is apparent that the shared cell types, specifically B and NK cell types, from both Mimitou_CITE_5k and Peterson_REAP_7k datasets have been merged together. However, it is noteworthy that cell types unique to each modality remain distinct and have not been fused together. We further validated scmFormer using a more challenging case, specifically by integrating two datasets, Mimitou_ASAP_5k and Mimitou_CITE_5k. In contrast to the previous example, these two datasets exhibit greater variations in sequencing technologies. However, scmFormer once again proves its capability by successfully integrating cell types from different datasets, with the exception of B cells (Figure 2d). These demonstrate that scmFormer satisfactorily removes the disparities introduced by diverse sequencing technologies, while simultaneously preserves inherent biological similarities across multi‐batch paired single‐cell datasets. Although some approaches also can integrate multi‐batch paired datasets, they exhibit unsatisfactory performance, except for scJoint that leverages prior cell type label information (Figures S8 and S9, Supporting Information).

#### 2.2.3 Integrating Triple‐Omics Data

Advances in sequencing technologies allow for simultaneous analysis of gene expression, chromatin accessibility and protein expression data.^[^ ^5^, ^54^, ^55^ ^]^ These technologies, coupled with decreasing sequencing costs in the future, will be wide‐spreadly adopted in comprehending cellular. Hence, we proposed an extension of scmFormer to handle triple‐omics data in a single step. Here, we take the Mimitou_DOGMA_14K dataset as an example. Figure 2e illustrates the remarkable capacity of scmFormer to seamlessly harmonize three disparate single‐cell omics data and enable the removal of technical noise while faithfully capturing essential biological information. Moreover, it is noteworthy that cells of the same type under various biological conditions manifest a remarkable proximity in the plot, indicating the ability of scmFormer in capturing subtle variations within the dataset during integrating process.

#### 2.2.4 Integrating COVID‐19 CITE‐Seq Datasets

**Figure 3:**
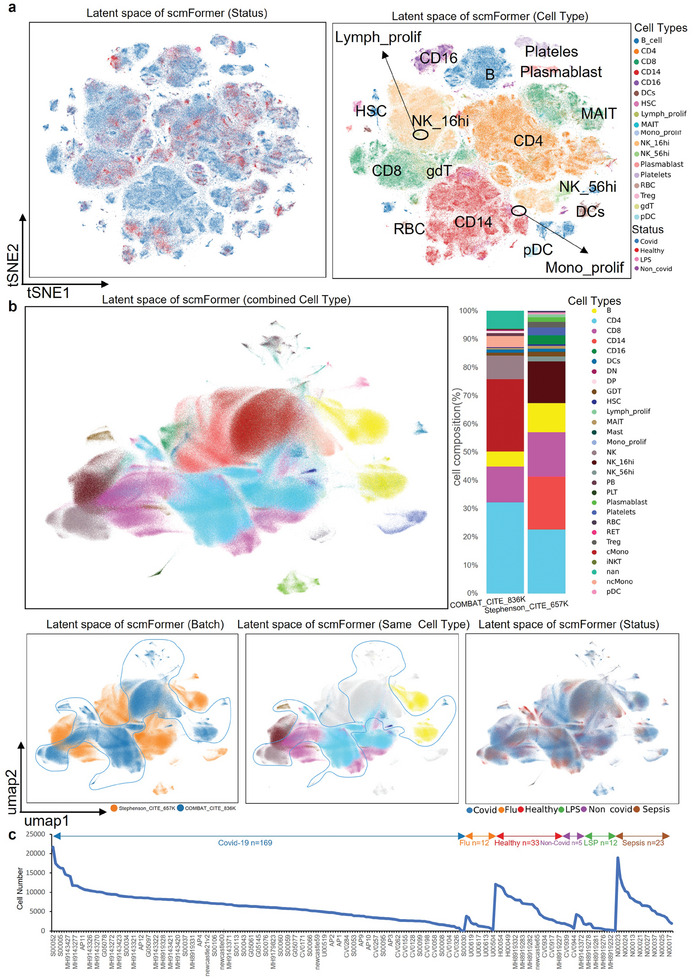
Integration of COVID‐19 cell atlas. a) tSNE (t‐Distributed Stochastic Neighbor Embedding) visualizations of the integrated latent space for all cells of Stephenson_CITE_657K, colored by donor status(left) and cell types(right). The Stephenson_CITE_657K dataset includes 102 COVID‐19 patients (527286 cells), 24 healthy individuals (97039 cells), 12 LPS‐labeled donors (7884 cells), and 5 asymptomatic COVID‐19 donors (15157 cells), representing four donor types. We also demonstrate the capacity of the scmFormer to process the COMBAT_CITE_836K, with its latent space depicted in the Figure S10 (Supporting Information). (b) tSNE visualizations of the latent space for all cells of Stephenson_CITE_657K and COMBAT_CITE_836K, colored by cell types(top), batch(bottom left), status batch(same cell types), status(bottom right). The integrated dataset comprises a total of 29 cell types, with 7 shared types including CD4, CD8, B cells, GDT, DCs, MAIT, and HSC. Unlike CD4 cells, which tend to aggregate, CD8 cells are dispersed, a phenomenon we attribute to the presence of distinct subtypes within the CD8 category (Figure S11, Supporting Information). c) Donor Status. The line graph shows 1483514 cells from 254 donors across six states: 169 with COVID‐19, 33 healthy, 12 LPS‐affected, 23 with spises, 12 with flu, and 5 asymptomatic COVID‐19 carriers.

To further substantiate the distinctive advantage of scmFormer in handling extensive datasets on regular laptops, we collected two COVID‐19 datasets (Stephenson_CITE_657K^[^ ^17^ ^]^ and COMBAT_CITE_836K^[^ ^16^ ^]^). Initially, we employed scmFormer to integrate data from diverse omics domains, enabling the generation of a more comprehensive cell atlas on a personal computer. The integration latent space provided by scmFormer revealed distinct cell types specific to COVID‐19 patients (**Figure** **3**a). Subsequently, we integrated these two large‐scale datasets, comprising a total of 1 483 514 cells, to provide a more comprehensive depiction of cell types. To address the challenge of noise in large‐scale datasets, which prevent cells of the same type from clustering together, we introduced linear optimal transport as a solution to this issue (Supplementary Method S2, Supporting Information). Consistent with the aforementioned validation, same cell types from different datasets exhibited convergence, which showed substantial dissimilarity due to disparate subpopulations across the datasets (Figure 3b; Figure S11, Supporting Information).

In general, by means of comparison and several case studies, scmFormer exhibits a notable proficiency in integrating single‐cell omics and proteomics data, surpassing other computational methods.

### 2.3 Generating Protein Data from Gene Expression Data

To address the substantial expenses associated with single‐cell multimodal sequencing, several methodologies have been devised to generate the unmeasured modality from the measured one.^[^ ^18^, ^31^, ^32^, ^33^, ^56^, ^57^, ^58^ ^]^ Hence, we propose the extension of scmFormer to tackle this problem effectively(Supplementary Method S3, Supporting Information).

**Figure 4:**
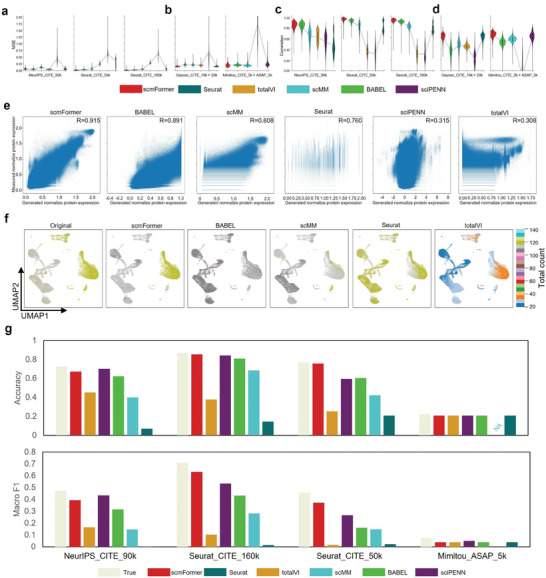
Performance of scmFormer on generating protein data from gene expression data. a‐d) Performance of methods measured by MSE (left) and Peason's correlation(right) on the five situation. e) Density scatterplot showing the expression of each gene in each cell within the test dataset of Seurat_CITE_160k (x axis represents generated normalize protein expression; y axis represents measured normalize protein expression). f) UMAP visualizations of generated normalize protein expression from Seurat_CITE_160k of each method, colored by total count. g) Performance of methods measured by accuracy on the four datasets.

In the initial investigation, we examined a straightforward scenario in which the training and testing datasets are derived from the same source but originate from different donors. In our experimental setup, we selectively allocate the data from two‐thirds of the donors for training, while the remaining one‐third are reserved for testing. In this investigation, we selected Seurat as well as a set of neural leaning‐based models including totalVI, scMM, BABEL, and sciPENN to conduct a thorough comparison. The evaluation encompassed three distinct datasets (NeurIPS_CITE_90k, Seurat_CITE_50k, and Seurat_CITE_160k). Prediction accuracy is assessed by calculating the Pearson's correlation and mean squared error (MSE). The performance of scmFormer exhibited superior performance compared to other methods, as demonstrated by the comparison results presented in **Figure** **4**a,c (Figure S12a and Table S1, Supporting Information).

In numerous instances, the training and testing datasets are typically derived from various sequencing platforms and occasionally aggregated from diverse tissue. Consequently, eliminating the batch effect within the various datasets poses a significant challenge when generating unmeasured modalities from the measured ones. To evaluate the efficacy of scmFormer, we implemented a “leave‐one‐dataset‐out” strategy on two distinct datasets. The first set of data, comprising Mimitou_CITE_5k and Mimitou_ASAP_5k, encompasses two different sequencing technologies: CITE‐seq and ASAP‐seq. The second datasets, consisting of Gayoso_CITE_20K and Gayoso_CITE_19K, encompasses two distinct tissues: spleen and lymph nodes. We conducted an evaluation of the performance of two pairwise train‐test combinations (Gayoso_CITE_19k > 20k, Mimitou_CITE_5k > ASAP_5k). In the term of MSE, scmFormer performs lower than the other method (Figure 4b). What's more, The results show that scmFormer exhibit significantly higher Pearson's correlation values compared to the other method (Figure 4d). Significantly, in the cross‐techniques scenario (Mimitou_CITE_5k > ASAP_5k), scmFormer demonstrated superiority over Seurat, substantiating its remarkable ability to effectively mitigate technique noise.

To provide a comprehensive comparison between scmFormer and other methods from multiple perspectives, we performed UMAP on the protein data generated by each method. Here, we take Seurat_CITE_160k as an example. Additionally, we employed a color scheme that represents the total count (Figure 4e,f). The UMAP plot effectively demonstrates that scmFormer is capable of generating protein profiles that closely resemble true protein expression levels, surpassing the performance of other models in this regard. Overall, across five datasets, scmFormer achieves notable performance improvements using the MSE metric on 80% (4 out of 5) of the benchmark datasets. Moreover, its MSE is only 0.2% shy of the second‐best reported value. In terms of Pearson's correlation, scmFormer surpasses all competing algorithms on each dataset and, on average, exhibits a 2% higher Pearson's correlation compared to the second‐best result (Table S1, Supporting Information).

To assess the effectiveness of the features of the generated modality data, we performed the cell‐type label transferring task. This task involved using the protein data from the training dataset as the reference dataset and the generated protein data as the query dataset. In the interest of fairness, we employed the K‐nearest neighbors (KNN) classifier as the classification method (Experimental Section). To establish a baseline, we initially employed the protein data of the test dataset as the query. As depicted in Figure 4g, scmFormer exhibited notably superior performance in terms of accuracy and F1‐score compared to other methods across the majority of datasets. Notably, the performance of the data generated by sciPENN was satisfactory in the term of classification; however, its generated data did not closely resemble the true data. Conversely, the data generated by Seurat did not present satisfactory classification results. scmFormer stands as the sole method capable of achieving a harmonious balance between both aspects while attaining optimal outcomes.

In general, scmFormer demonstrates competitive performance on the benchmarking datasets comparison to other methods. It is worth noting that scmFormer consistently performs well across various tasks involved in the analysis of single‐cell datasets multimodal containing a substantial number of cells. Leveraging the capabilities of scmFormer enables the generation of additional credible single‐cell multimodal data without incurring excessive costs.

### 2.4 scmFormer is Suitable for Spatial Multi‐Omics Data with Limited Cells

Spatial multi‐omics technologies offer distinct advantages over single‐cell multi‐omics approaches by elucidating spatial relationships, interactions, and localizations within the tissue, thereby providing a comprehensive understanding of spatial regulation in complex biological systems.^[^ ^59^, ^60^, ^61^, ^62^, ^63^ ^]^ Therefore, we extend scmFormer to analyze spatial multi‐omics.

#### 2.4.1 Generating Spatial Protein Data from Spatial Gene Expression Data

Spatial multi‐omics technologies suffer from inherent limitations, including lower resolution compared to single‐cell analysis, as well as the higher costs associated with their widespread adoption.^[^ ^48^, ^49^ ^]^ Consequently, we extended scmFormer to enable the generation of spatial protein data from spatial gene expression data. Notably, the limited cells (a few thousand cells) available for the training dataset poses an obstacle for deep learning‐based methods. We collected three groups benchmark datasets: SPACITE_Human_tissues (Human_skin_2K, Human_lung_2K, Human_spleen_2K, Human_thymus_2K, and Human_tonsil_2K)^[^ ^48^ ^]^ SPACITE_Mouse_tissues (Mouse_colon_2K, Mouse_intestine_1K, Mouse_kidney_2K, and Mouse_spleen_1K)^[^ ^48^ ^]^ and Xenium_tissues (Human_breast_4K, Human_brain_6K, and Human_tonsil_4K).^[^ ^49^ ^]^ The first and second group datasets were derived from Spatial CITE‐seq that enables high‐plex protein and whole transcriptome co‐mapping at a resolution of 20 µm.^[^ ^48^ ^]^ The third dataset was generated by 10x Genomics Xenium, a sequencing platform capable of detecting tissue spatial expression levels at subcellular resolution.^[^ ^49^ ^]^

**Figure 5:**
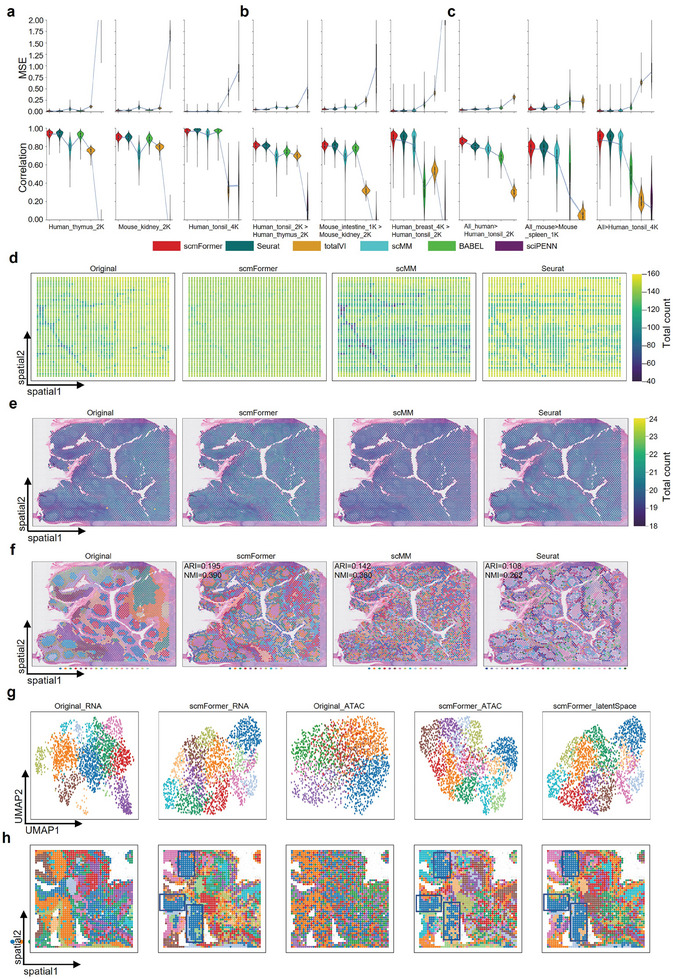
Performance of scmFormer on spatial multi‐omics. a–c) Performance of methods measured by MSE (top) and Peason's correlation(bottom) on the nine situation. d) Spatial visualizations of generated normalize protein expression from Human_tonsil_2K > Human_thymus_2K and (**e**) Human_breast_4K > Human_tonsil_2K of each method, colored by total count. f) Clustering performance of methods on the Human_tonsil_4K dataset. g) UMAP plots and h)Spatial plots were generated to visualize the clusters identified by Scanpy based on PCA of scRNA‐seq (first column) and scATAC‐seq data (third column), identified by Scanpy, utilizing the latent space of scmFormer for scRNA‐seq data (second column) and scATAC‐seq data (fourth column), identified by Scanpy in scmFormer's combined latent space (fifth column).

In the initial analysis, we considered a straightforward scenario where both the reference and query datasets are derived from the same datasets. We randomly select 80% of the cells as reference datasets and used the remaining 20% as query datasets. We select three datasets from the three group benchmark datasets (Human_thymus_2K, Mouse_kidney_2K, and Human_tonsil_4K). scmFormer exhibits superior performance compared to other methods (**Figure** **5**a; Figure 13a, Supporting Information). To examine the influence of tissue variations on the performance of the methods, we conducted cross‐tissue comparisons. We selected three pairwise train‐test combinations as benchmarking datasets (Human_tonsil_2K > Human_thymus_2K, Mouse_intestine_1K > Mouse_kidney_2K, Human_breast_4K > Human_tonsil_2K). These results, as depicted in Figure 5b, reveal that scmFormer slightly outperforms other methods in terms of MSE and Pearson's correlation (Figure S13b, Supporting Information). These suggest that scmFormer exhibits versatility in handling small‐scale datasets and is capable of mitigating tissue‐specific differences, enabling the generation of realistic protein profiles in real‐world scenarios (Figure 5d,e). To enhance the accuracy of generated unmeasured modalities and overcome limitations associated with a limited number of cells in a single reference dataset, we employed multi‐source datasets as the training dataset. We utilized three group datasets as benchmarking datasets (SPACITE_Human_tissues, SPACITE_Mouse_tissues, Xenium_tissues). Each dataset within the multiple datasets served as the query dataset, while the remaining datasets were combined to form the reference dataset. We selected three pairwise train‐test combinations to illustrate the results (All_human> Human_tonsil_2K, All_mouse>Mouse_spleen_1K, All>Human_tonsil_4K). scmFormer consistently outperformed other methods (Figure 5c; Figure S13c, Supporting Information), demonstrating its unique advantage in handling large‐scale datasets and mitigating disparities stemming from diverse data sources. To test the reliable of features generated by methods. The Human_tonsil_4K dataset underwent clustering analysis, resulting in the identification of 17 clusters (Figure 5f). Subsequently, we applied the same clustering approach to the protein data generated by each method under investigation. Remarkably, only the protein data generated by scmFormer demonstrated a clustering pattern that exhibited a high degree of similarity to the clustering observed in the true protein data. In contrast, methods such as Seurat and scMM exhibited limited proficiency in producing satisfactory clustering results, as evidenced by the results depicted in Figure 5f.

Overall, scmFormer demonstrates significant enhancements in performance, as measured by the MSE metric, across a total of nine benchmark datasets. Notably, it achieves superior MSE results on 89% (8 out of 9) of these datasets, falling behind the second‐best reported performance by a mere 1%. Additionally, scmFormer outperforms competing algorithms in terms of Pearson's correlation coefficient on 89% (8 out of 9) of the benchmark datasets, exhibiting an average Pearson's correlation coefficient that is 1% higher than the second‐best outcome (Table S2, Supporting Information).

#### 2.4.2 Integrating Spatial scATAC Data and Spatial Gene Expression Data

With the advancement of spatial multi‐omics technologies, several computational methodologies targeting spatial multi‐omics data are being developed.^[^ ^64^, ^65^ ^]^ In this section, we demonstrate the capability of scmFormer to integrate spatial multi‐omics data by adopting Zhang_Spatial ATAC‐RNA_2K as an example. scmFormer effectively eliminates noise and provides more reliable clustering outcomes, thus offering a valuable resource for downstream analyses. In comparison to the clustering results obtained after applying principal component analysis, the utilization of the latent space of scmFormer leads to more distinct clustering outcomes, particularly evident in the clustering of spatial scATAC‐seq data (Figure 5g). Furthermore, the clustering analysis reveals a disparity in the number of clusters obtained for spatial scRNA‐seq and scATAC‐seq data, which signifies the retention of modality‐specific information throughout the integration procedure. Furthermore, consistent cell clustering pattern are observed across scRNA‐seq, scATAC‐seq, and integrating data, underscoring the ability of scmFormer to retain shared information across different modalities (Figure 5h). With the aid of scmFormer, the analysis of spatial multi‐omics data, including cell type identification and the discovery of biological phenomena, is facilitated.

In summary, the results presented herein demonstrate the adaptability of scmFormer in the realm of spatial multi‐omics analysis. Notably, scmFormer exhibits exceptional performance not only on large‐scale datasets but also on mini‐scale datasets, surpassing neural network‐based algorithms and establishing itself as a formidable contender to the Seurat. These results underscore the wide‐ranging applicability of scmFormer and its potential to drive advancements in multi‐modal single‐cell analysis across diverse data scales.

## 3 Discussion

In recent years, the field of cellular biology has witnessed rapid progress owing to the development of high‐throughput single‐cell sequencing technologies.^[^ ^9^, ^12^, ^66^ ^]^ These technologies enable the simultaneous acquisition of diverse types of cellular data, such as genomics, transcriptomics, epigenomics, and proteomics. However, despite the development of numerous excellent methods designed for the integration of single‐cell multi‐omics data, integrating proteomics and other omics datasets has remained a challenging task, owing to the distinctive characteristics of single‐cell proteomics. To address this problem, we proposed a comprehensive and high‐performance multi‐task deep learning framework named scmFormer for integrating single‐cell multimodal data and generating unmeasured modality data from measured one.

This study presented a comprehensive evaluation of the scmFormer's ability to provide reliable latent representations for each modality and to enhance downstream analysis through the integration of multi‐omics data from diverse sources. We demonstrated that scmFormer offers a user‐friendly solution for handling diverse types of omics data. The evaluation and case studies reveal that scmFormer surpasses other methods across various aspect. First, scmFormer exhibits remarkable proficiency in harmonizing large‐scale single‐cell omics plus proteomics datasets at both the cell type and finer‐scale cell levels with limited computer resource. It is important to highlight that scmFormer stands apart from totalVI, specifically tailored for CITE‐seq analysis, because scmFormer present the exceptional ability to seamlessly integrate diverse omics data types and procure latent representations for distinct modalities. This provision not only simplifies downstream analysis but also enables insights that remain unattainable through the utilization of totalVI. Second, scmFormer possesses the capability to integrate multiple single‐cell paired multimodal datasets, leading to a dual benefit of reduced highly cost and enhanced biological insights. Third, scmFormer shows an outstanding ability to eliminate technical disparities between different omics modalities while preserving the underlying biological information inherent in the data, encompassing both cell types and experimental conditions. A noteworthy attribute of scmFormer lies in its ability to integrate extensive COVID‐19 datasets containing millions of cells, even under limited computational resources.

scmFormer transcends the limitation of solely integrating diverse datasets and also serves as a valuable tool for generating single‐cell multi‐omics data using existing data. This not only reduces the cost associated with sequencing but also augments the availability of data for in‐depth analysis of biological inquiries. Through a series of rigorous validations, scmFormer has demonstrated its exceptional capability to accurately generate single‐cell protein data from single‐cell gene expression data. Furthermore, we have also applied scmFormer to spatial multi‐omics data, demonstrating excellent performance even with limited data, surpassing other neural network‐based methods. This substantiates the notion that scmFormer is not only capable of handling large‐scale datasets but also excels in small‐scale datasets.

Existing single‐cell multimodal analysis models, although proficient in integrating multiple omics data types, predominantly focus on numerical data. However, with the rapid development of technologies, the utilization of image data has gained growing significance in the field of biology.^[^ ^67^, ^68^ ^]^ With the growing diversity of single‐cell omics data,^[^ ^69^, ^70^ ^]^ we believe that scmFormer will be well‐equipped to tackle the intricate challenges associated with integrating disparate data types in the field of omics.^[^ ^71^, ^72^ ^]^ Prior studies have successfully employed transformer‐based models pre‐trained on large‐scale scRNA‐seq data to enhance downstream tasks, emerging promising results.^[^ ^45^, ^46^ ^]^ Therefore, our future research endeavors will primarily focus on collecting a substantial volume of well‐annotated paired multi‐omics data and pretrain a single‐cell multi‐omics model. Additionally, drawing inspiration from the success of ChatGPT, we intend to incorporate reinforcement learning based on human feedback techniques in the pre‐training phase.^[^ ^44^ ^]^

The expanding scale of single‐cell multimodal datasets highlights the importance of accurate and efficient scmFormer for effectively extending its applications in real‐world scenarios. By effectively harmonizing various data modalities, scmFormer opens up new avenues for holistic analysis and interpretation of multi‐faceted biological phenomena at the single‐cell level.

## 4 Experimental Section

### The Overview of scmFormer

scmFormer is a transformer‐based tool structured by three modules, i.e., Cells Embedding, scTransformer Decoder Layer, and Linear Layer (Figure 1b).

### Cells Embedding

The scTransformer Decoder is designed to accept inputs in the form of real‐valued vectors. However, when considering single cell data, the inputs consist of individual expression values. In the case of scRNA‐seq data, an approach, introduced by CIForm, has been developed to partition gene expression directly into sub‐vectors(patches), which are subsequently fed into the Transformer Encoder^[^ ^36^ ^]^ Building upon the aforementioned research, a similar strategy was adopted by partitioning the gene (peak, protein) expression vector of each cell into uniform‐length sub‐vectors (patches), which are subsequently employed as inputs to scTransformer Decoder. This approach offers several benefits, including enhanced performance and reduced training time, as it mitigates noise arising from the transformation of individual gene (peak, protein) expression values into vectors within a single cell. Given that the treatment of different modalities follows a consistent procedure, now it would be focused on illustrating the application of the Cells Embedding module using gene expression data as an example.

Subsequent to the preprocessing of the gene expression matrix format within the single‐cell dataset, a series of operations were conducted to derive the sub‐vectors.

Let *X_c_* = (*X_c_*_,1,_ *X_c_*_,2,…,_ *X_c_*_,_ *_v_*) denote the gene expression vector of cell *c*, encompassing *v* highly variable genes (HVGs). To facilitate further analysis, the gene expression vector of cell *c* was partitioned into multiple sub‐vectors, each with a length denoted as *s*. The *i*‐th sub‐vector of cell *c* is denoted as *X_ci_* = (*X_c_*_,_ _i__*s+0,_ *X_c_*_,_ _i__*_ *_s_*_+1,_…_,_ *X_c_*_,_ _i*s+s‐i_), *i*∈(0,…,*v*|*s*), where *i* ranges from 0 to *v*|*s*. Moreover, the approach incorporates both encoded positional and categorical information into the input data stream to enhance model comprehension of the underlying structure. Specifically, “positional information” implies the sequential index of each sub‐vector within the entire array of sub‐vectors. It is crucial to note that this does not infer any hierarchical order based on gene variance but simply acknowledges the sequence arrangement. This sequential coding was embedded within each sub‐vector, reinforcing the model's ability to acknowledge and utilize the intrinsic order of the data, which was paramount since the sequence of sub‐vectors carries essential information that could significantly influence the final results. Regarding “categorical information,” this aspect serves a pivotal role in differentiating among various single‐cell modalities included in the input data. By way of illustration, for first modality, an encoded value of 1 was appended into every sub‐vector that belongs to it. Conversely, for second modality, an encoded value of 0 was introduced to each of its sub‐vectors. Such categorical encoding was instrumental in segregating and identifying data from different cell modalities, which was advantageous for maintaining the distinct attributes that are unique to each modality type.

### Implementation of the scTransformer Decoder Layer

To facilitate the analysis of single‐cell multimodal data, a scTransformer Decoder was designed (Figure 1c). The primary distinction lies in the incorporation of multiple rectified linear activation functions (ReLU) within the Transformer Decoder. The attention mechanism had played a significant role in the analysis of single‐cell transcriptomic data. Here, mask attention machine was adopted. Furthermore, the incorporation of ReLU was intended to facilitate more efficient extraction of features and enhance the learning of representations in the context of large‐scale single‐cell multimodal data analysis.

The attention network demonstrates exceptional performance owing to several notable properties that significantly contribute to its efficacy (Figure 1d). A salient property of the attention network is its capability to prioritize essential sub‐vectors within the expression vectors. This enables scmFormer to effectively disregard the influence of insignificant sub‐vectors, thereby mitigating their adverse impact on the overall analysis process. This characteristic empowers the attention network within scmFormer to exploit interdependencies across modalities, facilitating a more comprehensive integration and consolidation of information. Lastly, to generate the subsequent data, a masking mechanism is employed wherein all positions after the current position was masked by setting them to infinity. This precautionary measure prevented the model from “seeing” future information. As a result, the model can solely rely on previous value to generate the next value, thereby preserving the autoregressive nature of the process. This enhances the performance of scmFormer in generating unmeasured data by ensuring that the generation process strictly adhereed to the sequential dependencies and temporal coherence inherent in the data.

Besides, the utilization of multiple ReLU offers several advantageous features. First, ReLU is computationally efficient. Unlike more complex activation functions, such as sigmoid or tanh, ReLU only involved a simple thresholding operation, where negative inputs were directly set to zero. The computational efficiency of scmFormer allows it to handle large‐scale single‐cell multimodal data efficiently and to generate output that closely resembles the measured data. Another advantage of ReLU is its ability to introduce sparsity in activations. This sparsity can promote efficient information processing by encouraging scmFormer to focus on relevant features and disregarding irrelevant or noisy inputs introducing from various factor such as sequence technical, low‐confidence reads, batch effects and cell capture bias. Furthermore, ReLU helps scmFormer alleviate the overfitting problem due to preventing scmFormer from overemphasizing noise or outliers in the data. This regularization effect can enable scmFormer achieve amazing performance with limited spatial omic data.

Attention network in scmFormer is scm‐attention network, calculated by the following operation:

(1) $$\mathrm{scm}-\mathrm{attention}\;\left(Q,K,V\right)=\mathrm{ReLU}\left(\mathrm{Mask}\left(\frac{Q\times K^{T}}{\sqrt{s}}\right)\right)\times V$$

where,

(2) $$Q,K,V=\mathrm{ReLU}\left(\mathrm{split}\left(X\times W+b,s,\;\dim=2\right)\right)$$

where *X*∈ℝ^batch_size×v|s×s^ and *W, b*∈ℝ*^s^* ^×3^ *^s^*. The ReLU function is denoted as ReLU(*z_i_*) = max(*z_i_*,0). The resulting tensors *Q, K, V*∈ℝ^batch_szie×^ *^v^* ^|^ *^s^* ^×^ *^s^*.

For scmFormer, multi‐head self‐attention was used to capture different interaction information in multiple different projection spaces. The hyper parameter head was set to *H* in scmFormer, which means that *H* had parallel self‐attention operations, i.e.,

(3) $$\mathrm{MulitHead}\left(Q,K,V\right)=\mathrm{Concat}\;\left(\mathrm{hea}d_{1},\text{…}\text{…},\mathrm{hea}d_{H}\right)$$

where,

(4) $$\mathrm{hea}d_{h}=\mathrm{scm}-\mathrm{attention}\;\left(Q_{h},K_{h},V_{h}\right)$$

After layer normalization and multi‐head attention,

(5) $$X_{\mathrm{scm}-\mathrm{attention}}=X+\mathrm{MulitHead}\left(\mathrm{LayerNorm}\left(X\right)\right)$$

*X* is converted into *X_scm‐_*_attention_ ∈ℝ^batch_size×v|s×s^ as the input of the feed‐forward network. $\mathrm{LayerNorm}\left(z_{i}\right)=\frac{z_{i}-\mu }{\sigma }\times \gamma +\beta$, where *z_i_* is the *i*‐th element of the input vector *z*, *µ* is the mean of *z*, *σ* is the standard deviation of *z*, and *γ* and *β* are learnable parameters.

The scm‐attention network is useful for capturing dependencies between elements among sub‐vectors. However, it may not be enough to capture all non‐linear features among sub‐vectors. To address this, the feed‐forward network is added. Feed‐forward network consists of two linear layers, with a ReLU between them, i.e.

(6) $$\begin{array}{ccc} X_{\mathrm{decoder}} & = & X_{\mathrm{scm}-\mathrm{attention}}+Re\mathrm{LU}\left(\mathrm{LayerNorm}\left(X_{\mathrm{scm}-\mathrm{attention}}\right)W_{1}+b_{1}\right)W_{2}\\ & & +b_{2} \end{array}$$

where *X_decoder_* ∈ℝ^batch_size×v|s×s,^ with *W*_1_, *b*_1_∈ℝ^s∙2s^ and *W*_2_, *b*_2_∈ℝ^2s∙s^ representing the linear projection weights.

### Implementation of the Linear Layer

Preceding the Linear Layer, the application of an average pooling layer is employed to compute the mean value across all sub‐vectors.

(7) $$X_{\mathrm{average}}=\mathrm{mean}\left(X_{\mathrm{decoder}}\right)$$

where *X*_average_∈ℝ^batch_size×^ *^s^*.

One linearly connected networks was then used to reconstruct the cell:

(8) $$X_{\mathrm{reconstruction}}=X_{\mathrm{average}}W_{1}+b_{1}$$

where *X*_reconstructede_∈ℝ^batch_size×v^ and *W*_1_, *b*_1_∈ℝ^s×v^.

### Multi‐Task Learning

To achieve a closer resemblance of the reconstructed data to the measured data, data recovery task was employed and adopt MSE loss function. Within this function, *X* represents the measured cell data, while *X*_reconstruction_ represents the output generated by scmFormer:

(9) $$\mathrm{Los}s_{\mathrm{rec}}=MSE\left(X_{\mathrm{reconstruction},}X\right)$$

In order to facilitate an improved fusion of different modalities of data, data fusion task was employed and adopted the MSE loss function. Within this context, *X*_average_mod1_ represents the latent representation of the first single‐cell modality, while *X*_average_mod2_ represents the latent representation of the second single‐cell modality:

(10) $$\mathrm{Los}s_{\mathrm{comb}}=MSE\left(X_{\mathrm{average}\_\mathrm{mod}1},X_{\mathrm{average}\_\mathrm{mod}2}\right)$$

where *X*_average_mod1_, *X*_average_mod2_∈ℝ^batch_size×s|2^ are paired.

By utilizing the MSE loss, it was aimed to minimize the discrepancy between the average values of the two modalities, thereby enhancing the integration and alignment of the different modalities within the model.

In the context of integrating single‐cell multimodal data, the following loss function was employed:

(11) $$\mathrm{Los}s_{\mathrm{integration}}=\lambda \times \mathrm{Los}s_{\mathrm{rec}}+\mathrm{Los}s_{\mathrm{comb}}$$

where λ is a parameter adjusting the weight of Loss_rec_.

With this particular design, it was aimed to preserve both the shared information across different modalities and retain the modality‐specific information. By incorporating this approach, strived to strike a balance that allows for the preservation of common features while also capturing the distinctive characteristics inherent to each modality.

For the task of generating the unmeasured modality, the following loss function was employed:

(12) $$\mathrm{Los}s_{\mathrm{generating}}=\mathrm{Los}s_{\mathrm{rec}}$$

### Training Details

For the integration of multimodal single‐cell data, the training was conducted using a fixed learning rate of 1e‐4. The batch size used for most tasks was set to 32. For further details on hyperparameters, please refer to Table S3 (Supporting Information). Moreover, to thoroughly evaluate the robustness of the scmFormer parameters, a series of experiments was conducted using several representative datasets. These tasks included “Integrating NeurlPS_Multiome_70k,” “Generating NeurlPS_Multiome_70k,” “All_human > Human_tonsil_2K,” “Human_tonsil_2K > Human_thymus_2K,” and “Generating Human_tonsil_4K.” The parameters tested were HVGs, length of sub‐vectors, λ, epoch, number of heads, drop rate, learning rate, and batch size, as detailed in the Supporting Information (Figures S14 and S15, Supporting Information). Overall, it is observed that variations in the epoch and number of heads had minimal impact on model performance. For paired dataset integration tasks, there was a decrease in performance as the drop rate increased. On the other hand, performance changes were negligible for other tasks. In the “Generating Human_tonsil_4K” task, increasing the number of HVGs resulted in reduced model efficiency, while the impact on other tasks was once again minimal. The lambda parameter demonstrated a normal distribution from 1 to 600, with optimal performance observed within the range of 40–100. Across all tasks, the learning rate showed little effect on model performance within a specific range (0.00001–0.005). Similarly, the model performance remained stable across a wide batch size range (from 2 to 2048). Regarding the length of sub‐vectors, adhering was recommended to the established parameters and consulting the robustness tests provided, as the number of proteins imposes constraints on this parameter.

The experimental environment utilized in the study comprised a 12th Generation Intel(R) Core(TM) i7‐12700H processor running at 2.30 GHz and an NVIDIA GeForce RTX 3050 Laptop GPU with 16GB of memory and 453GB of storage.

### Metrics for Evaluation

The evaluation of cell‐type label transferring accuracy is performed using two metrics: the macro F1 score and the accuracy score. In this study, a kNN classifier was provided by the sklearn. The training process was based on cell‐type annotations and reference data, such as the latent representation of scRNA‐seq data. Subsequently, the well‐trained kNN classifier was applied to predict cell‐type annotations of a query dataset, which consisted of scATAC's latent representation. The accuracy and macro F1 scores were then computed by comparing the real and predicted cell type annotations of the query dataset.

The FOSCTTM metric is employed to assess the preservation of relationship in cell‐to‐cell pairings' neighborhood. This evaluation was conducted on two datasets where the cell‐to‐cell correspondence information was known. To calculate FOSCTTM, the following formula was utilized:

(13) $$\mathrm{FOSCTTM}=\frac{1}{2n}\left(\sum_{i=1}^{N}\frac{n_{1}^{\left(i\right)}}{N}+\sum_{i=1}^{N}\frac{n_{2}^{\left(i\right)}}{N}\right)$$

(14) $$n_{1}^{\left(i\right)}=\left|\left\{j|d\left(x_{j},y_{i}\right)<d\left(x_{i},y_{j}\right)\right\}\right|$$

(15) $$n_{2}^{\left(i\right)}=\left|\left\{j|d\left(x_{i},y_{j}\right)<d\left(x_{i},y_{i}\right)\right\}\right|$$

where the $n_{1}^{\left(i\right)}$, and $n_{2}^{\left(i\right)}$ represent the counts of cells in the first and second datasets, respectively, that exhibit closer proximity to the *i*‐th cell than their respective true matches in the opposing dataset. The Euclidean distance, denoted as *d*, is utilized as a measure of proximity between cells. It is important to note that the FOSCTTM metric is bounded within the range of 0 to 1, where lower values signify higher accuracy in preserving the relationship between cell‐to‐cell pairings.

Pearson's correlation, Spearman's correlation, RMSE, and MSE was used to evaluate the quality of generated data.

### Data and Pre‐processing

To comprehensively evaluate the effectiveness of scmFormer, a systematic assessment was conducted using a collection of twenty‐four benchmark datasets in the field of single‐cell analysis. These datasets encompassed diverse characteristics, including two different species (human and mouse), thirteen distinct tissues (retina, PBMC, skin, BMMC, spleen, lymph nodes, thymus, tonsil, colon, intestine, kidney, breast, brain), and eleven varied sequencing protocols (CITE‐seq, ASAP‐seq, REAP‐seq, Spatial ATAC–RNA‐seq, Spatial‐CITE‐seq, 10x Genomics Xenium, DOGMA‐seq).

For scRNA‐Seq data, the total counts of each cell were normalized and log‐normalizatized using the Scanpy. As for the protein data, the total counts of each cell were normalized using the centered log‐ratio using Muon^[^ ^73^ ^]^ To align the dimensions of the scRNA‐Seq data and protein data, principal component analysis (PCA) was employed.

### Tools for Comparison

A meticulous benchmarking study was conducted to assess the performance of scmFormer against several methods. In the R environment, the efficacy of scmFormer was evaluated in comparison to Seurat, Liger, iNMF, BindSC, and Harmony. Likewise, within the Python environment, scmFormer against scVI, Pamona, UnionCom, scJoint, totalVI, BABEL, scMM, and sciPENN was scrutinized. For a detailed description of the methodology, please refer to the Supplementary Note S1 (Supporting Information).

## Conflict of Interest

The authors declare no conflict of interest.

## Author Contributions

X.Z. and J.X. conceived and designed this project; J.X. designed and implemented the computational framework and conducted benchmarks and case studies with guidance from X.Z; J.X., X.Z., and D.S.H. wrote the manuscript and gave some insightful suggestions. All authors read and approved the manuscript.

## Supporting information

Supporting Information

## Data Availability

The data that support the findings of this study are available in the supplementary material of this article.
