# Supplementary material for: scmFormer Integrates Large‐Scale Single‐Cell Proteomics and Transcriptomics Data by Multi‐Task Transformer
Source: Adv Sci (Weinh). 2024 Mar 14;11(19):2307835. doi: 10.1002/advs.202307835 (PMC11109621; doi:10.1002/advs.202307835)
Supplement: Supplementary file 1 — Supporting Information [file ADVS-11-2307835-s001.pdf]

ADVANCED SCIENCE
Open Access

Supporting Information

for *Adv. Sci.*, DOI 10.1002/advs.202307835

scmFormer Integrates Large-Scale Single-Cell Proteomics and Transcriptomics Data by Multi-Task Transformer

*Jing Xu, De-Shuang Huang and Xiujun Zhang**

Supplementary Information

scmFormer integrates large-scale single-cell proteomics and transcriptomics data by multi-task transformer

Jing Xu[1,2], De-Shuang Huang[4] , Xiujun Zhang[1,3*]

1 Key Laboratory of Plant Germplasm Enhancement and Specialty Agriculture, Wuhan Botanical Garden, Chinese Academy of Sciences, Wuhan 430074, China

2 University of Chinese Academy of Sciences, Beijing 100049, China

3 Center of Economic Botany, Core Botanical Gardens, Chinese Academy of Sciences, Wuhan, 430074 China

4 Eastern Institute for Advanced Study, Eastern Institute of Technology, Ningbo, China

* Corresponding author: Xiujun Zhang, Key Laboratory of Plant Germplasm Enhancement and Specialty Agriculture, Wuhan Botanical Garden, Chinese Academy of Sciences, Wuhan 430074, China. Tel: 86-027-87700844, Fax: 86-027-87700844, Email: zhangxj@wbgcas.cn.

Supplementary Figures

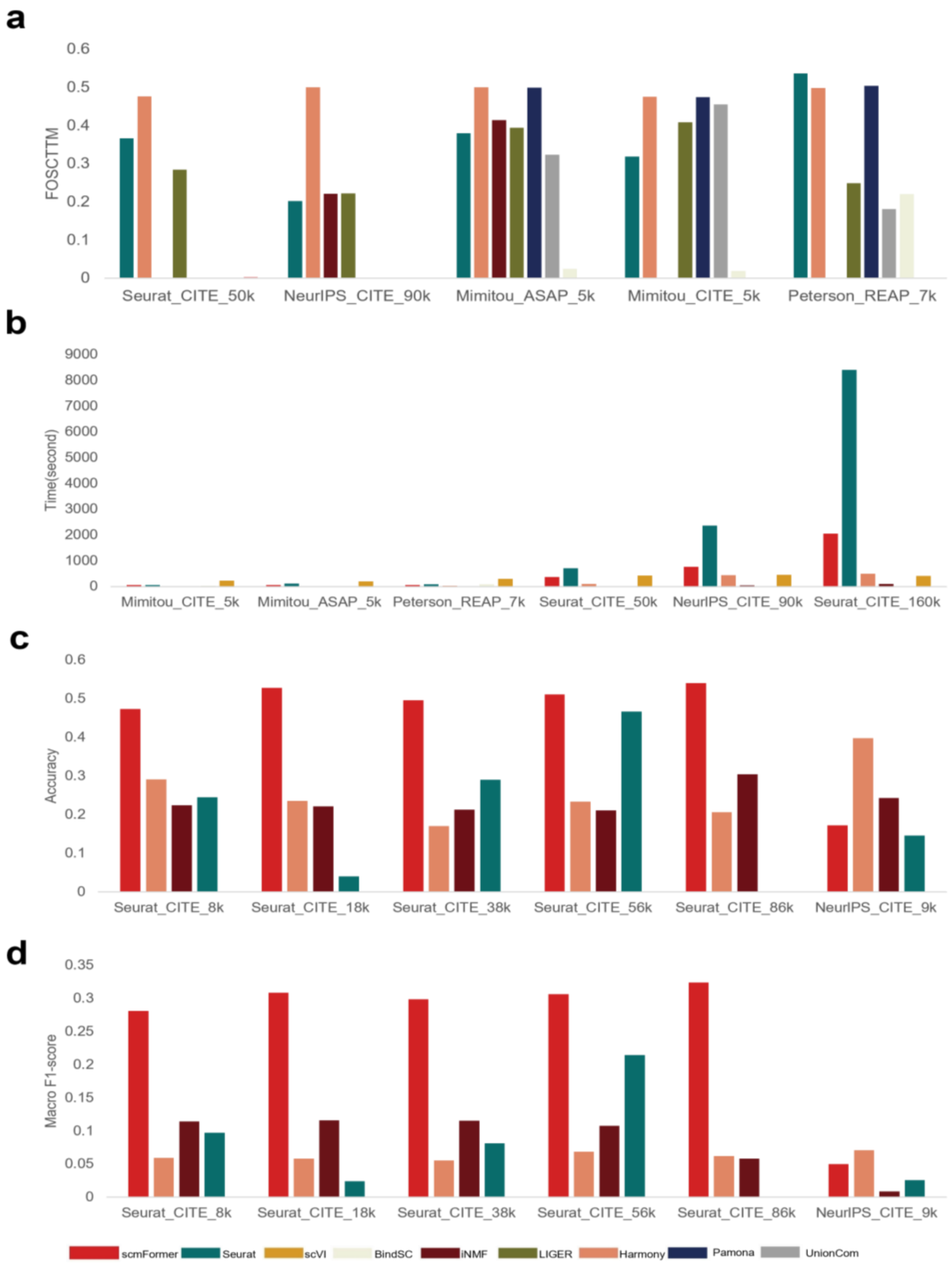

Figure S1: Performance of methods on integration scRNA-seq and protein data. (**a**) Performance of methods in the term of FOSCTTM. (**b**) Running time of methods. (**c-d**) Performance of scmFormer on integration unpaired scRNA-seq and protein data in terms of accuracy and macro F1.

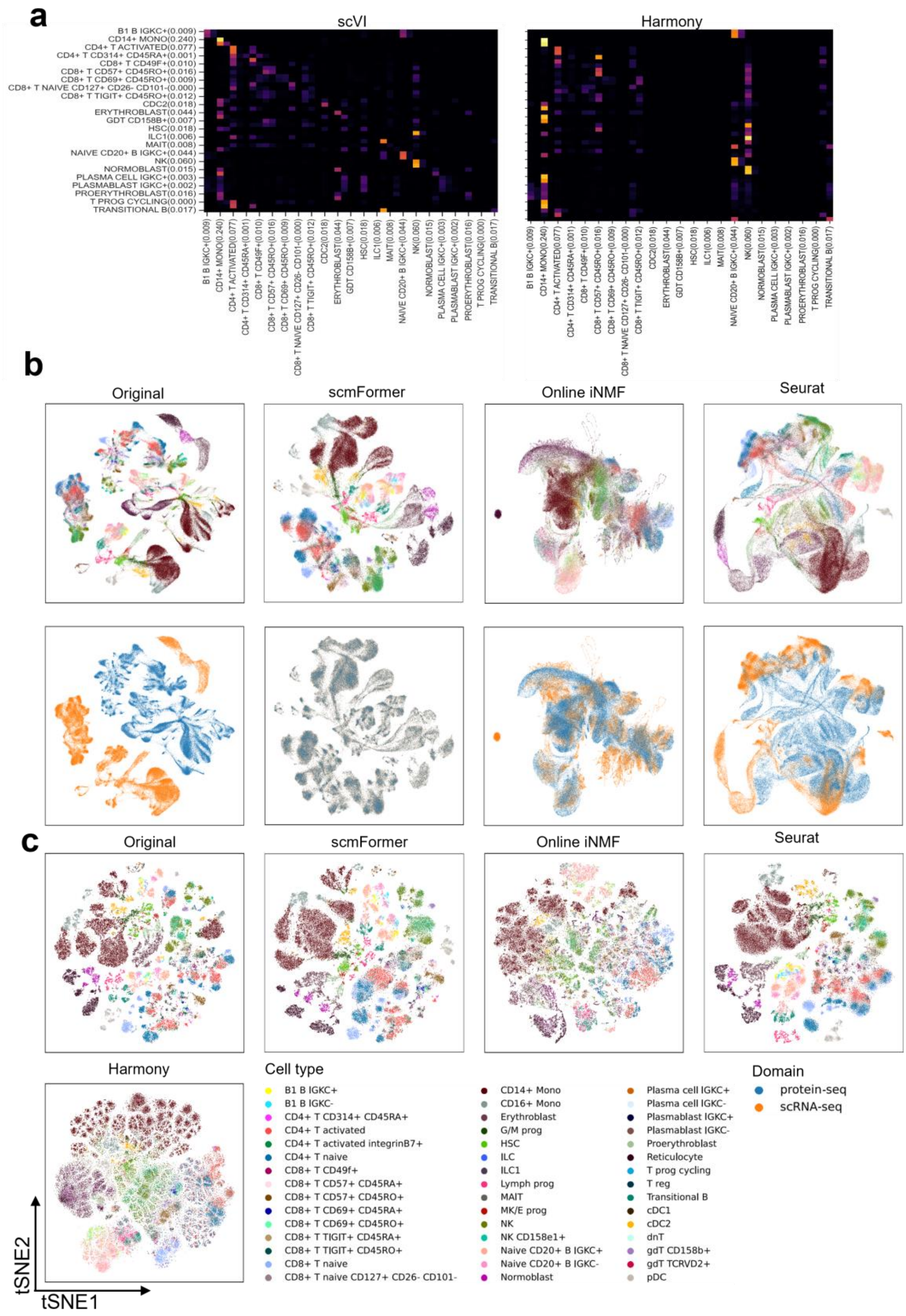

Figure S2: Performance of methods on integration the NeurIPS_CITE_90k dataset.

(**a**) Confusion matrix heatmaps for cross-validation results of Harmony and scVI on

the largest dataset (NeurIPS_CITE_90k dataset). (**b**) UMAP visualizations of the cell embeddings of different modalities in the NeurIPS_CITE_90k dataset aligned with different integration methods. (**c**) tSNE visualizations of the cell embeddings of integrated modalities in the NeurIPS_CITE_90k.

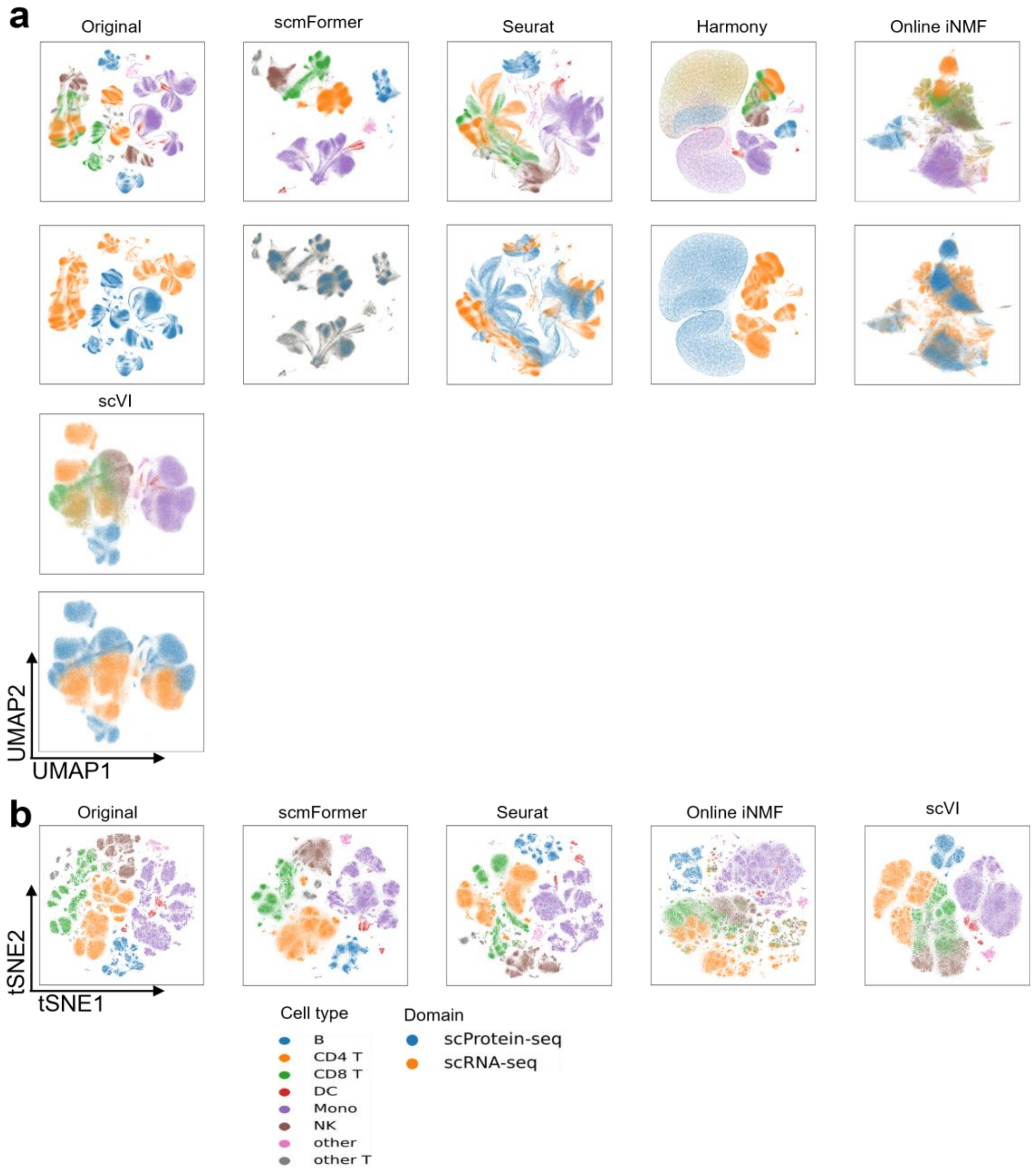

Figure S3: Performance of methods on integration Seurat_CITE_160k dataset. (**a**) UMAP visualizations of the cell embeddings of different modalities in the Seurat_CITE_160k dataset aligned with different integration methods. (**b**) tSNE visualizations of the cell embeddings of integrated modalities in the Seurat_CITE_160k dataset.

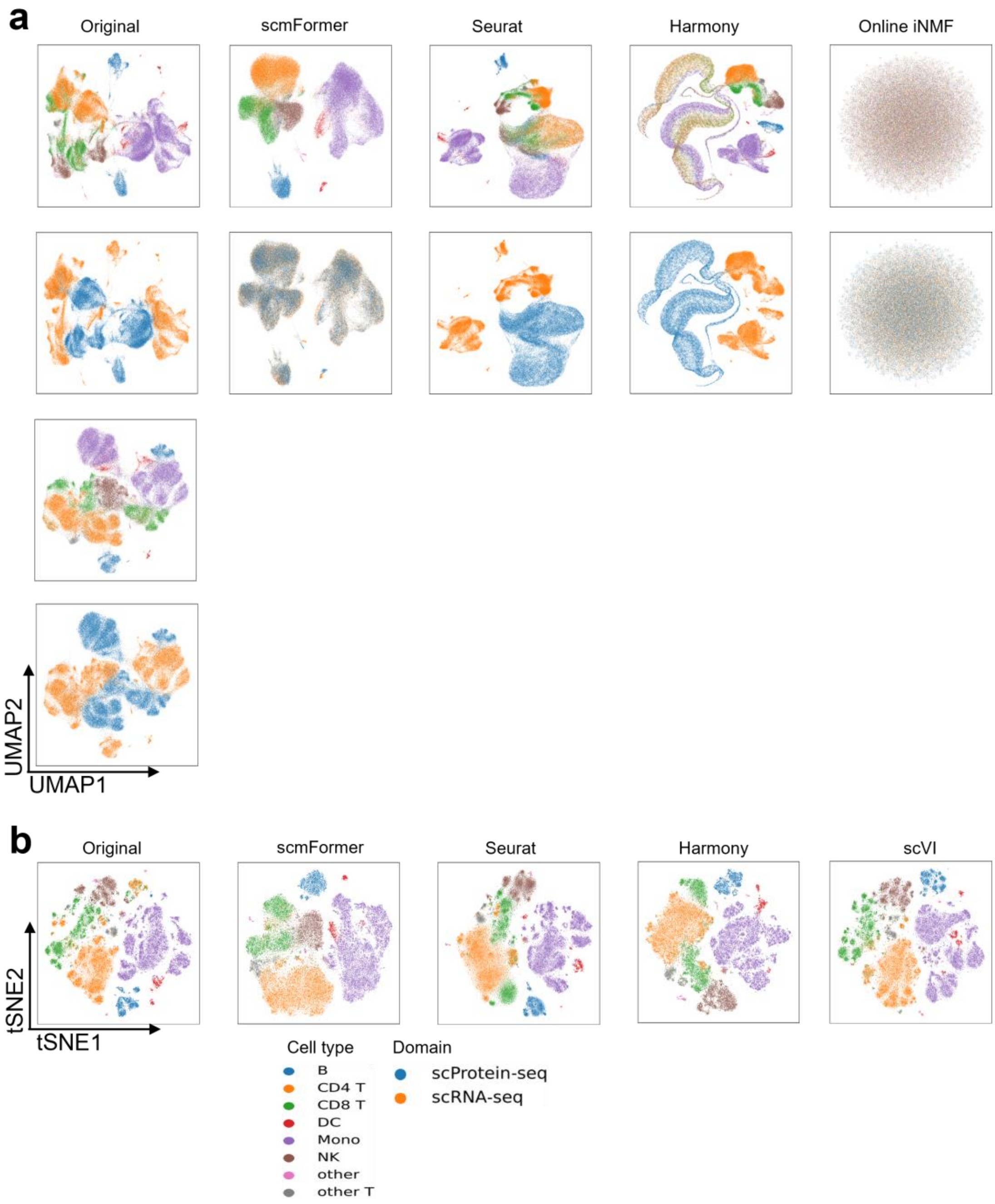

Figure S4: Performance of methods on integration Seurat_CITE_50k dataset. (**a**) UMAP visualizations of the cell embeddings of different modalities in the Seurat_CITE_50k dataset aligned with different integration methods. (**b**) tSNE visualizations of the cell embeddings of integrated modalities in the Seurat_CITE_50k dataset.

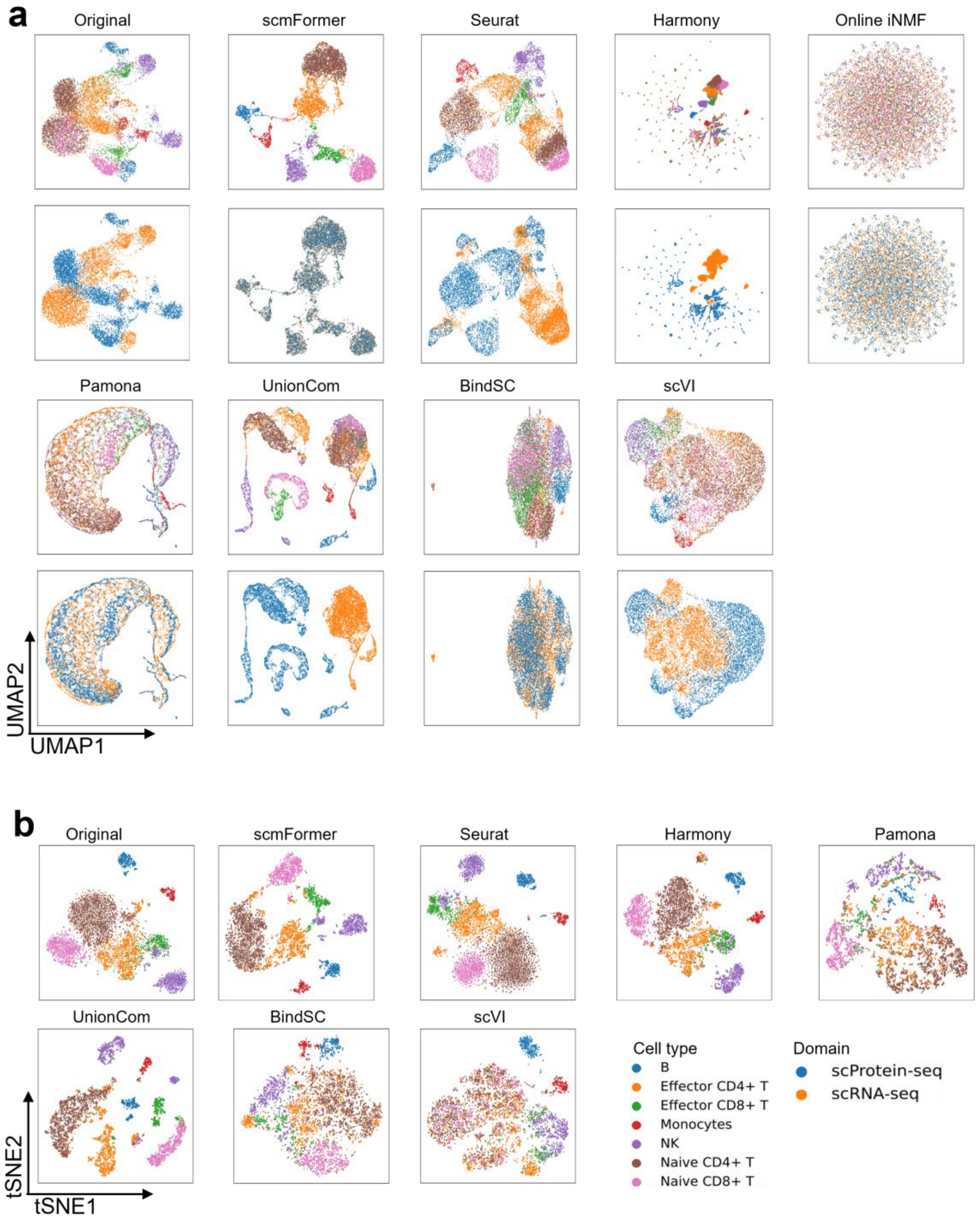

Figure S5: Performance of methods on integration Mimitou_CITE_5k dataset. (**a**) UMAP visualizations of the cell embeddings of different modalities in the Mimitou_CITE_5k dataset aligned with different integration methods. (**b**) tSNE visualizations of the cell embeddings of integrated modalities in the Mimitou_CITE_5k dataset.

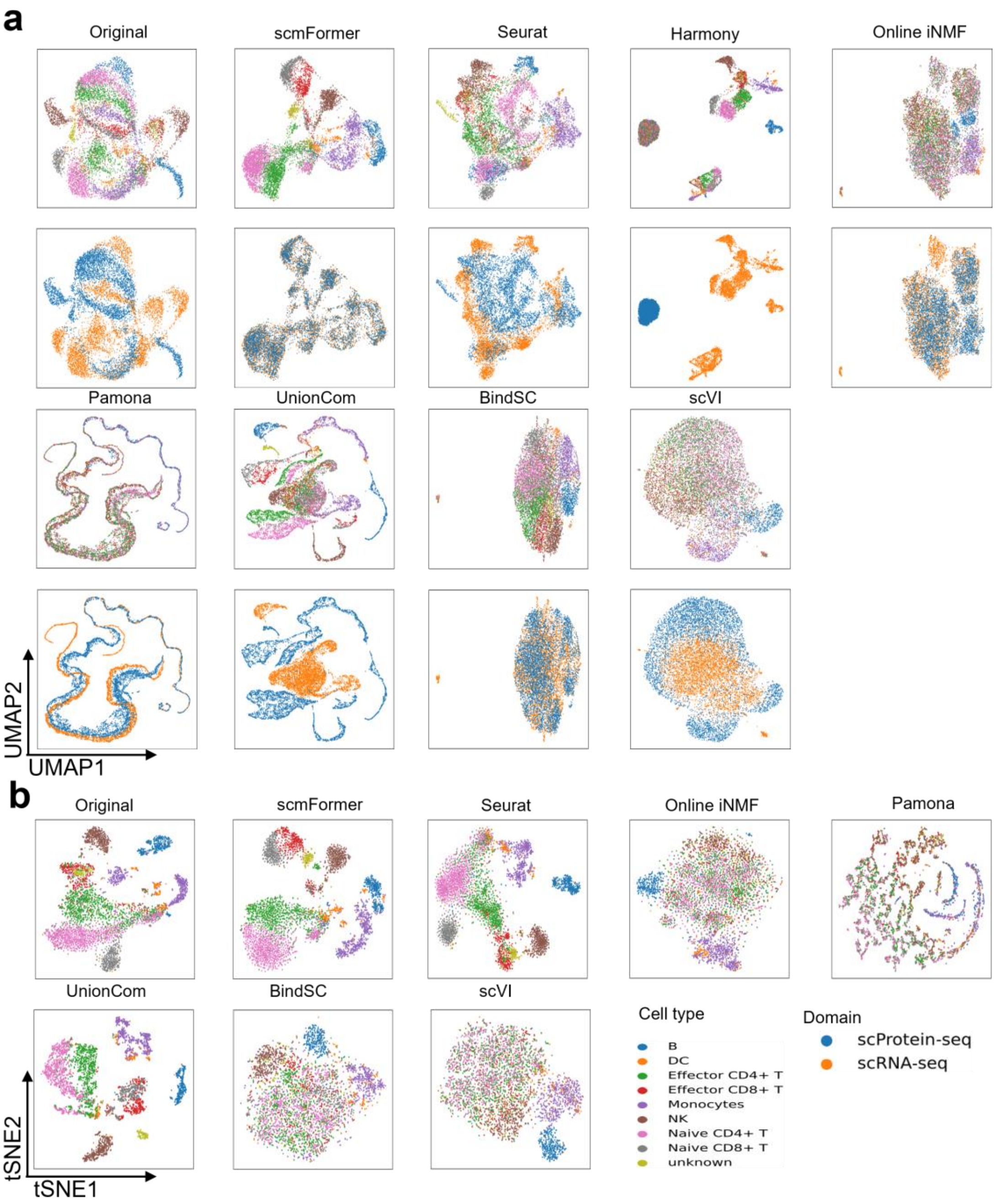

Figure S6: Performance of methods on integration Mimitou_ASAP_5k dataset. (**a**) UMAP visualizations of the cell embeddings of different modalities in the Mimitou_ASAP_5k dataset aligned with different integration methods. (**b**) tSNE visualizations of the cell embeddings of integrated modalities in the Mimitou_ASAP_5k dataset.

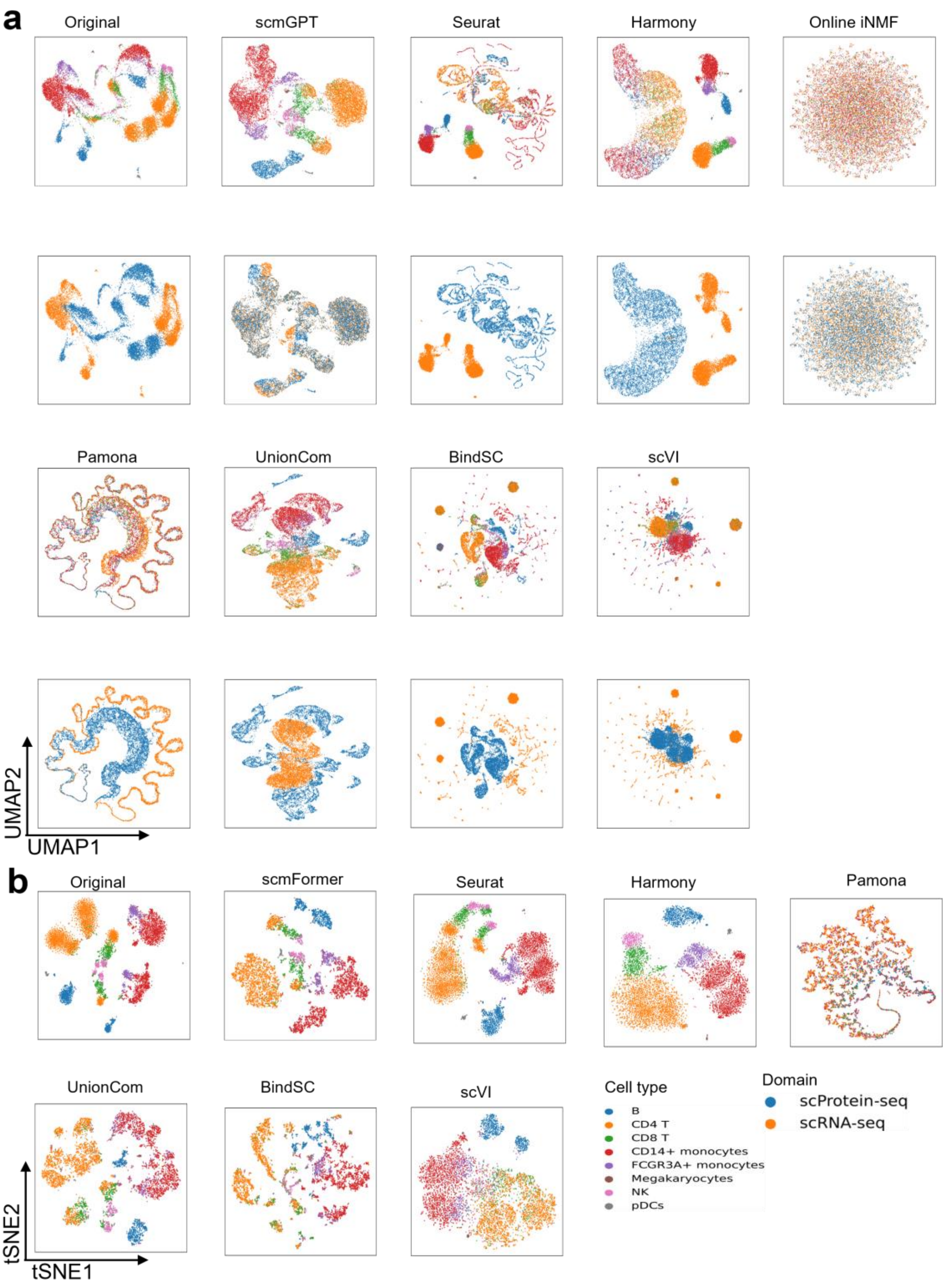

Figure S7: Performance of methods on integration Peterson_REAP_7k dataset. (**a**) UMAP visualizations of the cell embeddings of different modalities in the Peterson_REAP_7k dataset aligned with different integration methods. (**b**) tSNE visualizations of the cell embeddings of integrated modalities in the Peterson_REAP_7k dataset.

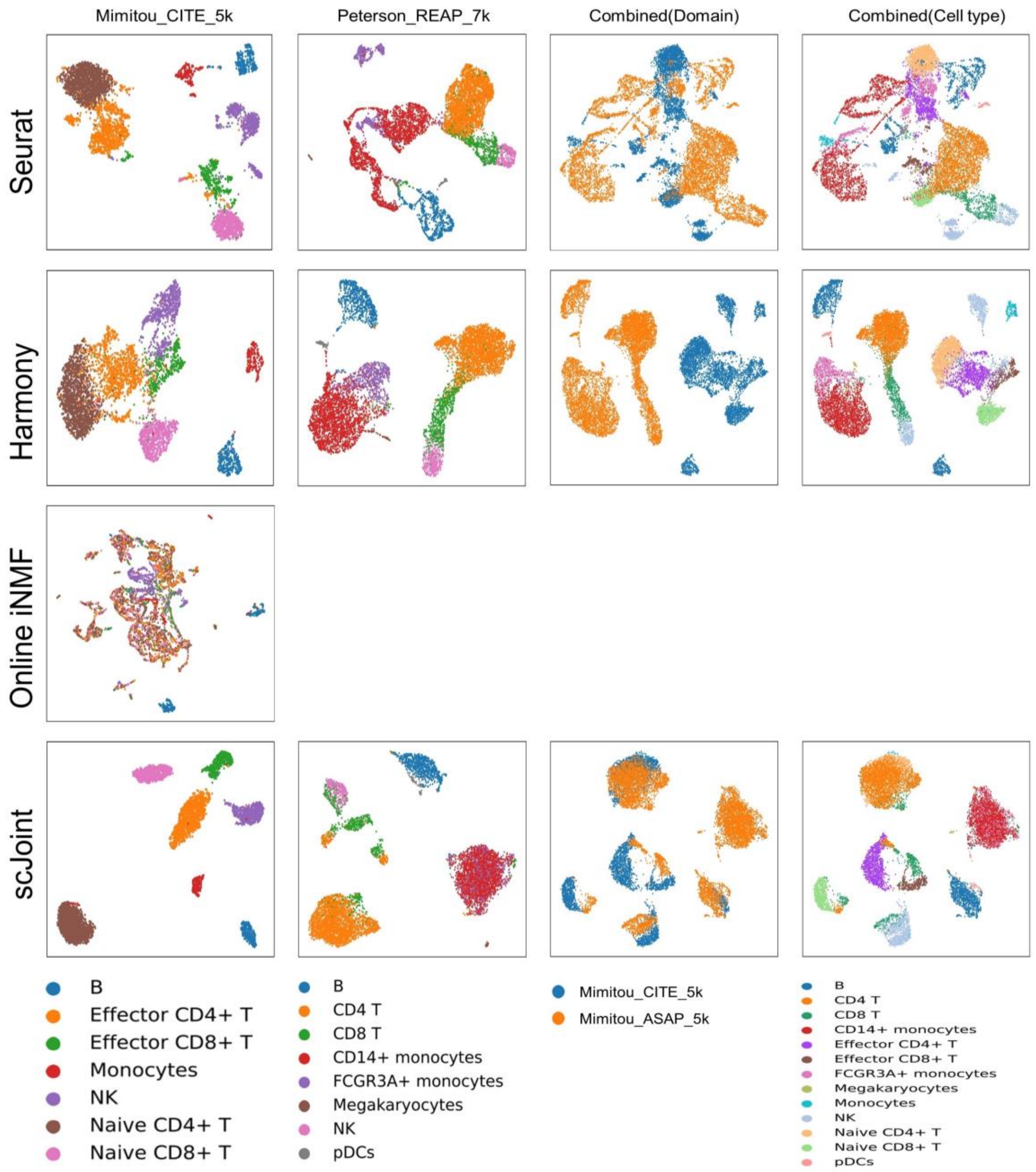

Figure S8: Performance of methods on integration the Mimitou_CITE_5k and Peterson_REAP_7k.

UMAP visualizations of the cell embeddings for all cells of Mimitou_CITE_5k (first column) and Peterson_REAP_7k (second column), colored by cell types. UMAP visualizations of the integrated cell embeddings for all cells of Mimitou_CITE_5k and Peterson_REAP_7k, colored by omics layers(third column) and cell types(fourth column).

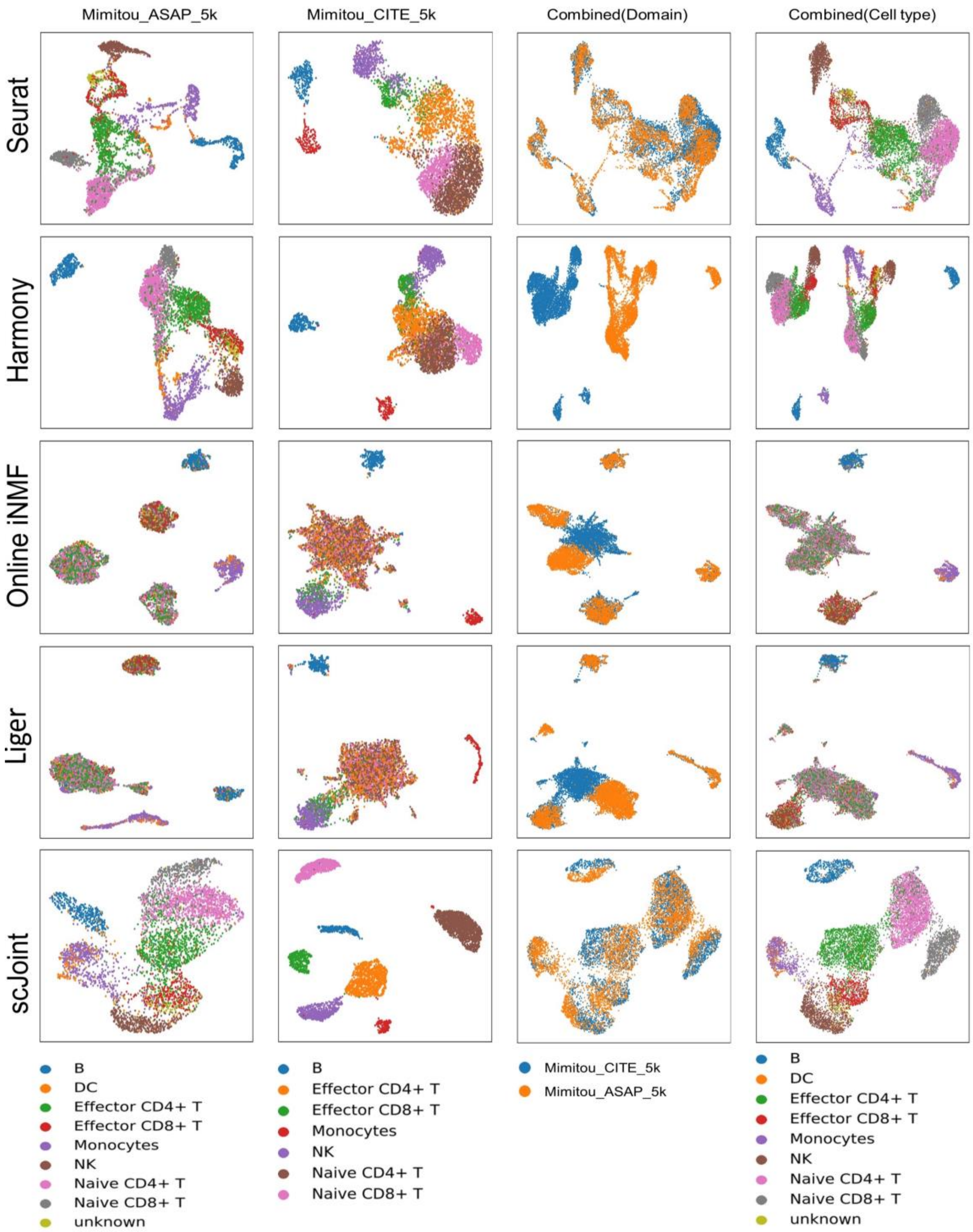

Figure S9: Performance of methods on integration the Mimitou_CITE_5k and Mimitou_CITE_5k.

UMAP visualizations of the cell embeddings for all cells of Mimitou_ASAP_5k (first column) and Mimitou_CITE_5k (second column), colored by cell types. UMAP visualizations of the integrated cell embeddings for all cells of Mimitou_ASAP_5k and Mimitou_CITE_5k, colored by omics layers (third column) and cell types(fourth column).

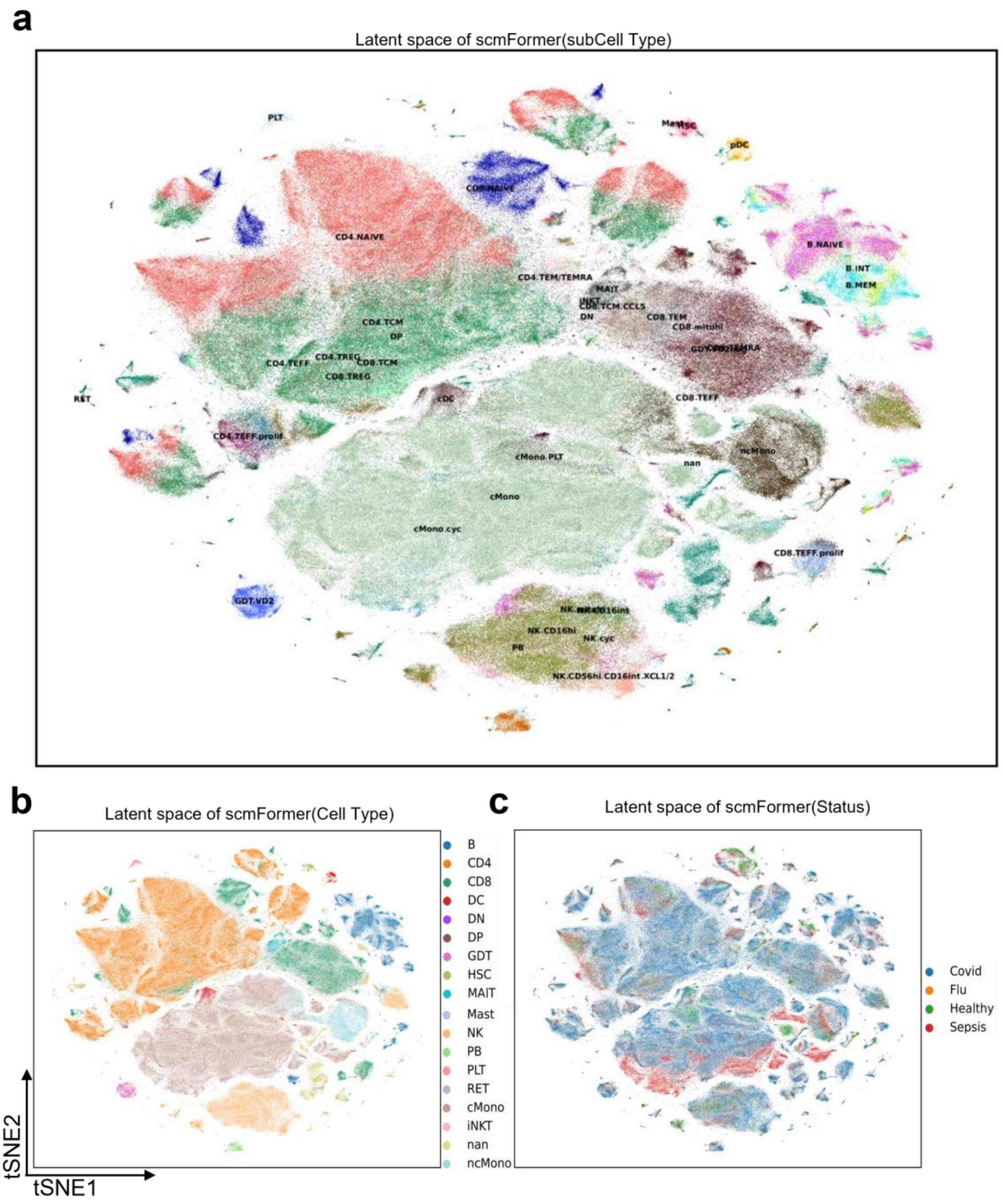

Figure S10: Integration of COMBAT_CITE_836K datasets.

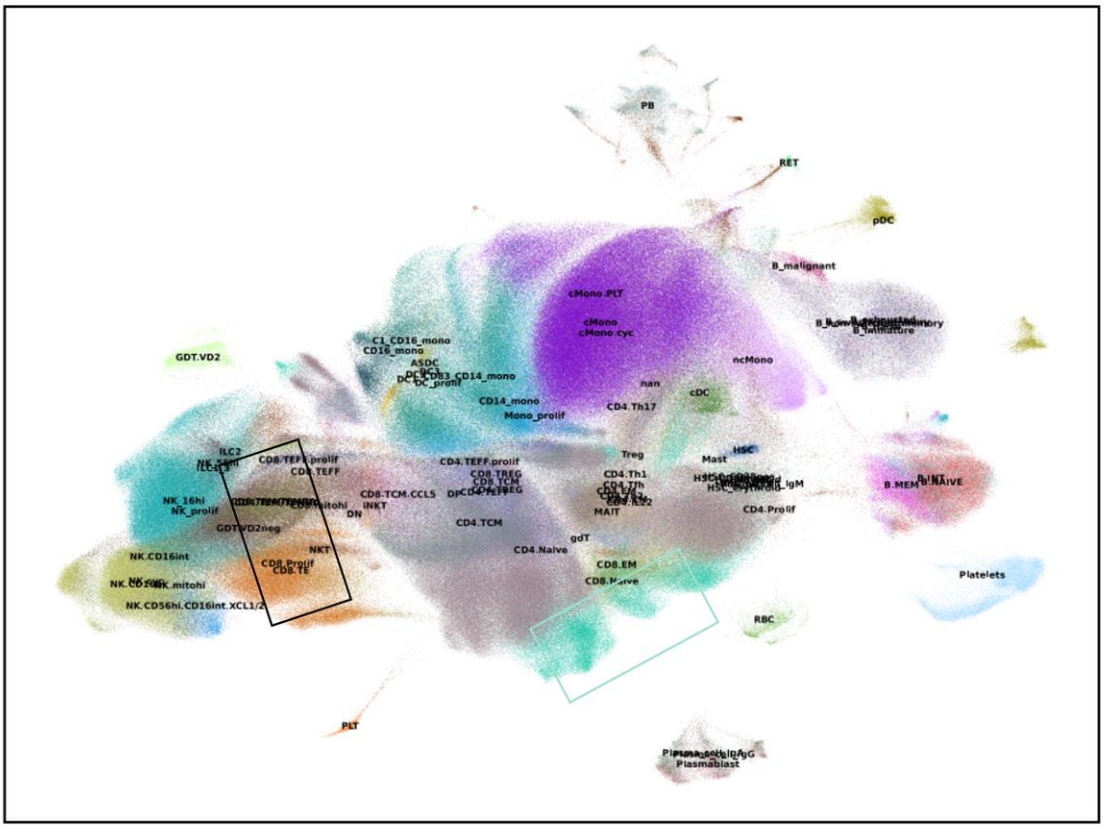

Figure S11: subCellType of Integration of Stephenson_CITE_657K and COMBAT_CITE_836K datasets.

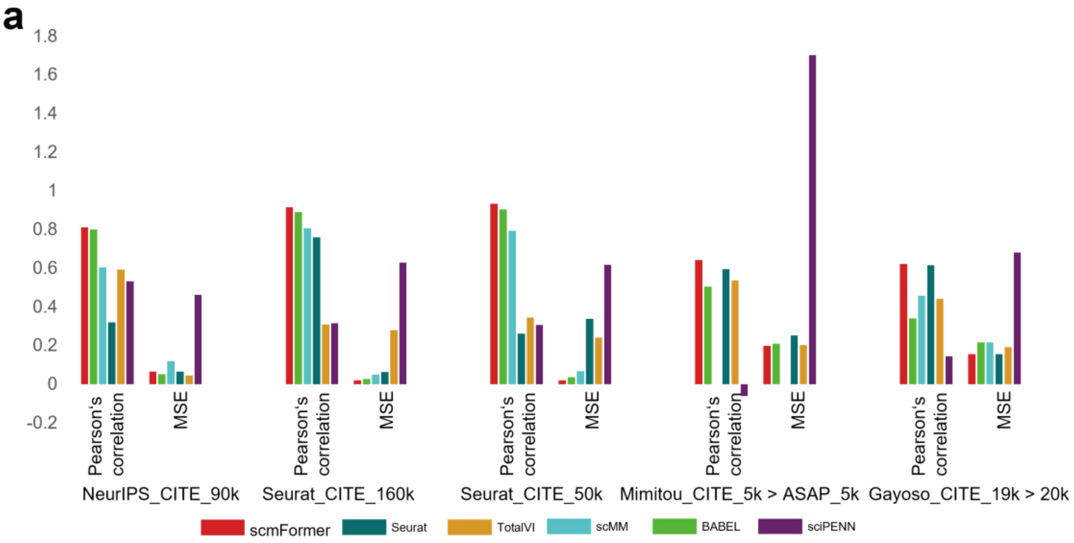

Figure S12: Performance of scmFormer on generating protein data from gene expression data.

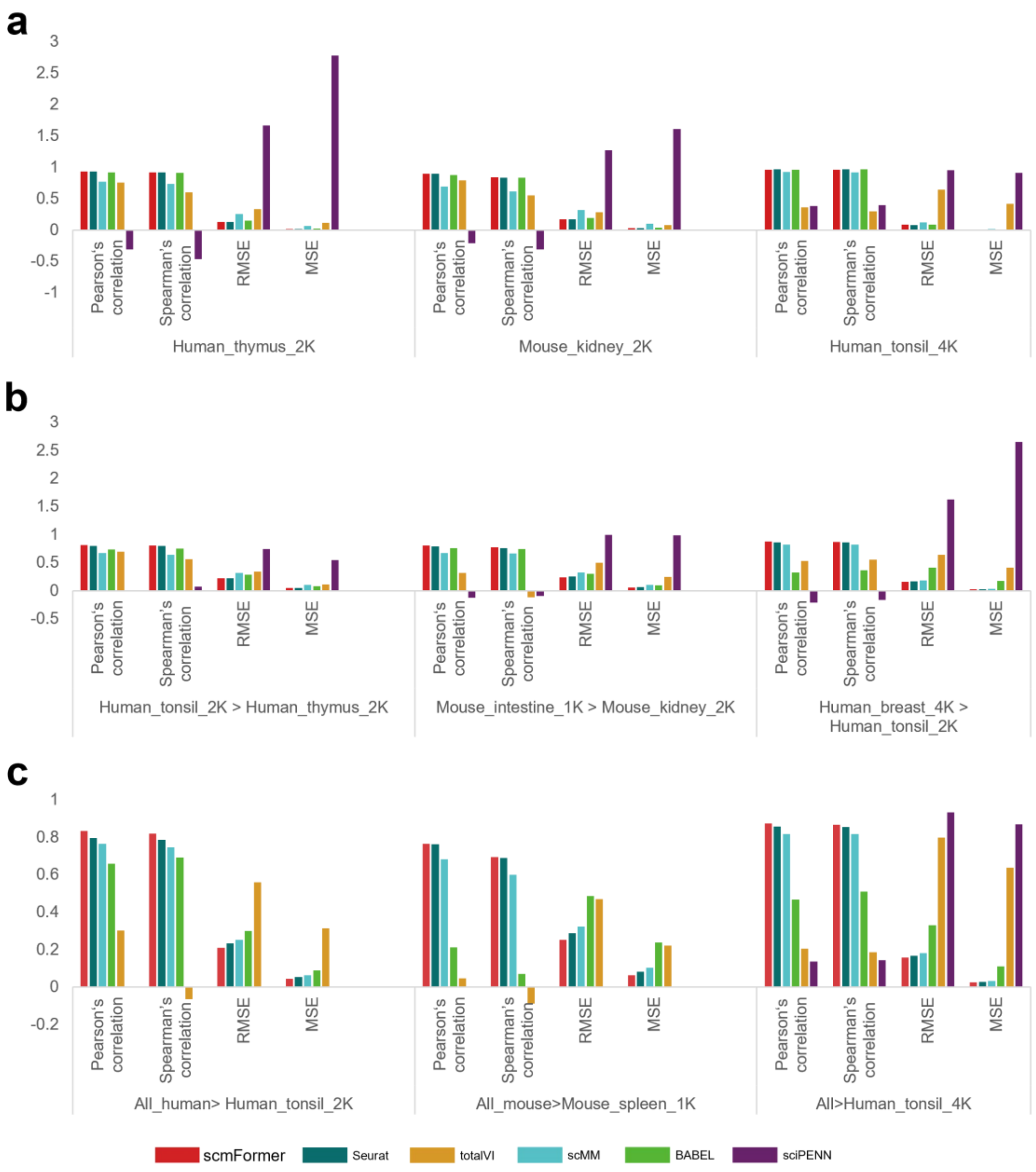

Figure S13: Performance of scmFormer on generating spatial protein data from spatial gene expression data.

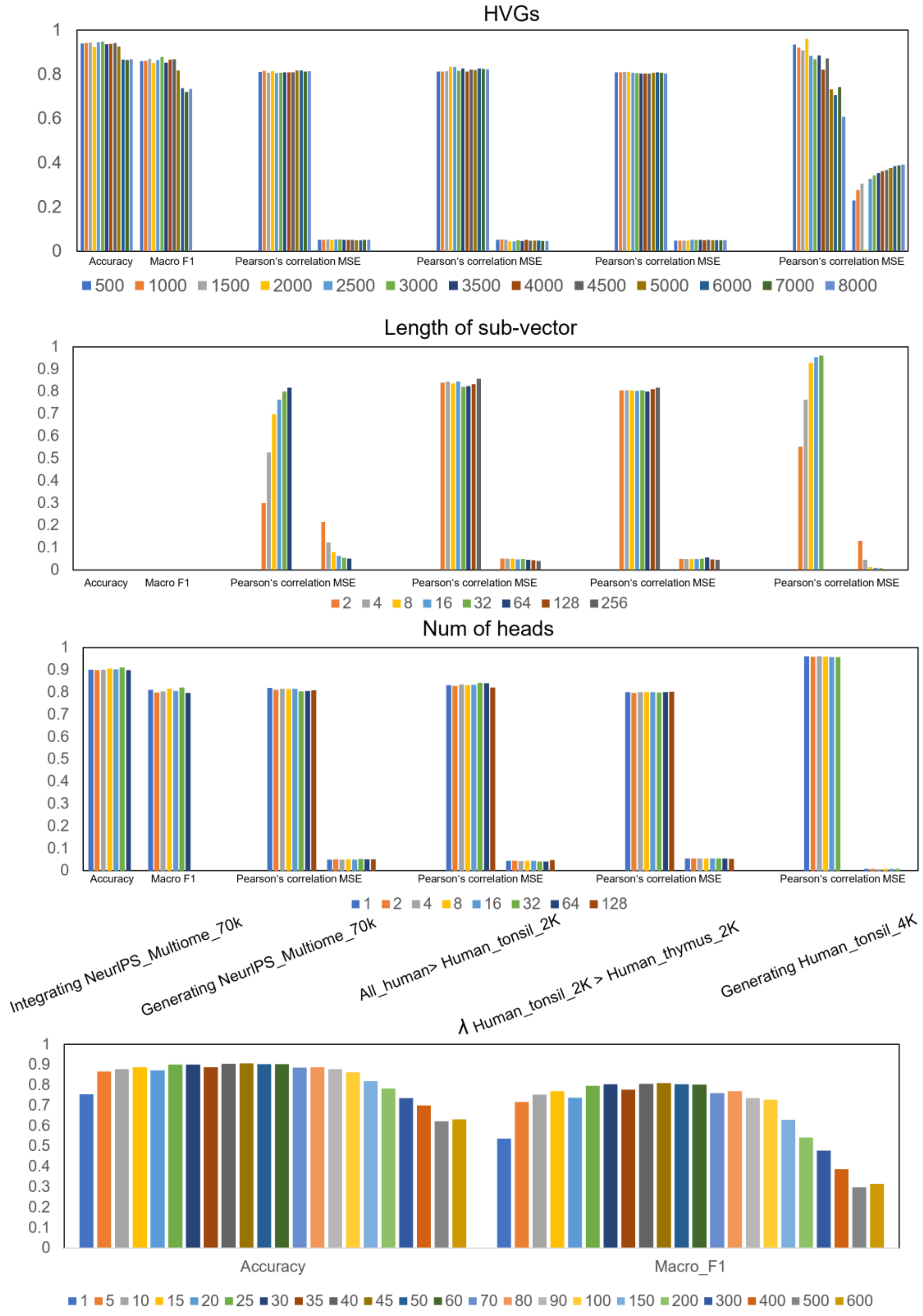

Figure S14. Robustness of scmFormer.

We conducted robustness testing on three hyperparameters(HVGs, Length of sub-vector, Num of heads across six datasets. The parameter λ is utilized to balance the trade-off between two different loss functions. However, in the task of generating

unmeasured single-cell modalities, there is only a single loss component, rendering the λ parameter unnecessary. Consequently, we have conducted λ robustness experiments exclusively on integration tasks, where the interplay of multiple loss functions demands such fine-tuning.

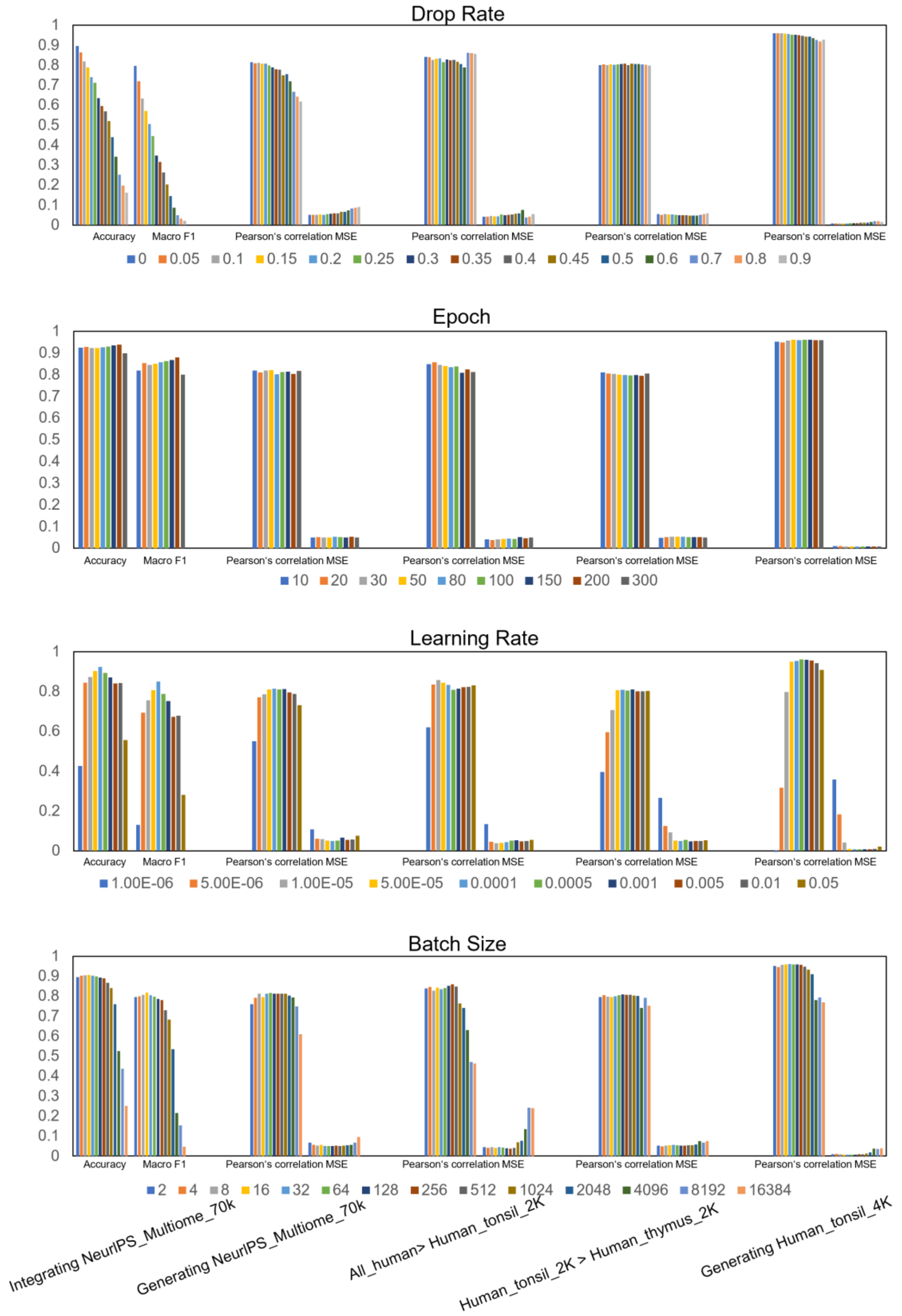

Figure S15. Robustness of scmFormer.

We conducted robustness testing on four hyperparameters(Drop Rate, Epoch, Learning Rate, Batch Size) across six datasets.

Table S1. Performance of scmFormer on generating protein data from gene expression data.

	**NeurIPS_CITE_90k**		**Seurat_CITE_160k**		**Seurat_CITE_50k**		**Mimitou_CITE_5k > ASAP_5k**		**Gayoso_CITE_19k > 20k**	
	Pearson‘s correlation	MSE	Pearson‘s correlation	MSE	Pearson‘s correlation	MSE	Pearson‘s correlation	MSE	Pearson‘s correlation	MSE
scmFormer	**0.812**	0.066	**0.915**	**0.02**	**0.933**	**0.022**	**0.642**	**0.198**	**0.622**	**0.15**
BABEL	0.801	0.052	0.891	0.03	0.904	0.037	0.505	0.21	0.341	0.22
scMM	0.606	0.12	0.808	0.05	0.794	0.068	Na	Na	0.457	0.22
Seurat	0.319	0.065	0.76	0.06	0.262	0.338	0.596	0.253	0.616	0.16
TotalVI	0.594	**0.044**	0.308	0.28	0.346	0.241	0.537	0.203	0.443	0.19
sciPENN	0.533	0.461	0.315	0.63	0.306	0.618	-0.06	1.702	0.144	0.68

Table S2. Performance of scmFormer on generating spatial protein data from spatial gene expression data.

	**Human_thymus_2K**		**Mouse_kidney_2K**		**Human_tonsil_4K**	
	Pearson‘s correlation	MSE	Pearson‘s correlation	MSE	Pearson‘s correlation	MSE
scmFormer	**0.934**	**0.016**	**0.895**	**0.029**	0.960	0.008
Seurat	0.934	0.016	0.893	0.029	**0.968**	**0.006**
scMM	0.772	0.065	0.69	0.1	0.923	0.014
BABEL	0.915	0.022	0.872	0.04	0.959	0.008
TotalVI	0.752	0.112	0.788	0.08	0.361	0.416
sciPENN	-0.31	2.779	-0.21	1.61	0.381	0.909

	Human_tonsil_2K > Human_thymus_2K		Mouse_intestine_1K > Mouse_kidney_2K		Human_breast_4K > Human_tonsil_2K	
	Pearson's correlation	MSE	Pearson's correlation	MSE	Pearson's correlation	MSE
scmFormer	**0.810**	**0.047**	**0.804**	**0.06**	**0.877**	**0.024**
Seurat	0.8	0.05	0.793	0.06	0.864	0.027
scMM	0.675	0.101	0.669	0.11	0.819	0.034
BABEL	0.736	0.081	0.761	0.09	0.321	0.171
TotalVI	0.697	0.114	0.317	0.25	0.528	0.414
sciPENN	-0	0.546	-0.13	0.98	-0.21	2.656

	All_human> Human_tonsil_2K		All_mouse>Mouse_spleen_1K		All>Human_tonsil_4K	
	Pearson's correlation	MSE	Pearson's correlation	MSE	Pearson's correlation	MSE
scmFormer	**0.856**	**0.039**	**0.763**	**0.064**	**0.873**	**0.025**
Seurat	0.796	0.054	0.762	0.08	0.858	0.028
scMM	0.766	0.064	0.682	0.1	0.818	0.033
BABEL	0.66	0.09	0.213	0.24	0.467	0.109
TotalVI	0.303	0.314	0.047	0.22	0.205	0.638
sciPENN	Na	Na	Na	Na	0.136	0.87

Table S3. Hyper-parameters of scmFormer.

As noted, scmFormer has a total of nine parameters, which fall into three categories: data preprocessing parameters, model parameters, and training parameters. Here is a breakdown of each along with the settings applied across various datasets:

1. **Data Preprocessing Parameters:**
   - The number of top genes: 2000 for all datasets (default 2000).
   - Length of sub-vectors, number of principal components: These vary

based on the specific integration task and the number of protein in the scProtein data when integrating single-cell proteomic and transcriptomic data. For integrating single-cell epigenomic and transcriptomic data, the length of sub-vectors is 128, and the number of heads is 8.
  - The number of principal components for all data generation tasks is determined by the smallest number of features in the two modalities being integrated.

2. **Model Parameters:**
   - λ: This parameter balances the two loss functions. It is set to 50 when involving two modalities and 100 for three modalities (default 50).
   - Number of heads: Adjusted based on the number of protein or as specified in the preprocessing parameters.
   - Drop rate: 0 for all integration tasks (default 0,0.05,0.1).
3. **Training Parameters:**
   - Epochs: Set to 50 for most tasks, except for the inner-dataset generation task for 'Generating protein data from gene expression data', where it is set to 10 (default 50).
   - Learning rate: 0.0001 for all integration works.(default 0.0001 or 0.001)
   - Batch size: 32 for most tasks, but reduced to 4 when integrating spatial scATAC and scRNA data (default 32).

A detailed table of parameter settings for different datasets is provided below. The table will serve as a reference for researchers aiming to replicate our findings or extend the scmFormer methodology to new datasets. We believe that these details will contribute to the transparency and reproducibility of our work.

**Integrating Task**	Integrating scRNA-seq and scProtein	Mimitou_DOGMA_14k	Zhang_Spatial ATAC-RNA_2K	**Mulit-Batch paired datasets Integration Task**	Mimitou_CITE_5k and Peterson_REAP_7k	Mimitou_ASAP_5k and Mimitou_CITE_5k	Stephenson_CITE_657K and COMBAT_CITE_836K
HVGs	2000			HVGs	2000		
Length of sub-vectors	#	#	128	Length of sub-vectors	16	192	64
Num of principal component	#	#	#	Num of principal component	40	216	100

λ	50	100	50	λ	50	100	50
Num of head	#	#	8	Num of head	4	32	16
Drop Rate	0			Drop Rate	0		
Epoch	50			Epoch	50		
Learning Rate	0.0001			Learning Rate	0.0001		
Batch Size	32	32	4	Batch Size	32		

The integration of scRNA-seq and scProtein data involves determining the length of sub-vectors, the number of principal components, and the number of heads based on the number of proteins measured in scProtein-seq data. The details are as follows in the table below:

Num of protein	<=40	40-50	50-100	100-200	200-300
Number of principal component	30	40	50	100	200
Length of sub-vectors	16	32	32	64	192
Number of head	2	2	8	16	32

**Generating Task1 inner**	NeurIPS_CITE_90k	Seurat_CITE_50k	Seurat_CITE_160k	Human_thymus_2K	Mouse_kidney_2K	Human_tonsil_4K
HVGs	2000					
Length of sub-vectors	128			256	128	32
Num of principal component	Number of principal component is based on the number of proteins measured in scProtein-seq data.					
λ	50					

Num of head	16	64	64	16
Drop Rate	0.05	0.1	0.1	0
Epoch	10	50		
Learning Rate	0.001	0.0001	0.0001	0.001
Batch Size	32			

**Generating Task2 cross**	Gayoso_CITE_19k > 20k	Mimitou_CITE_5k > ASAP_5k	Mouse_intestine_1K > Mouse_kidney_2K	All_mouse>Mouse_spleen_1K	All_human> Human_tonsil_2K	Human_tonsil_2K > Human_thymus_2K	Human_breast_4K > Human_tonsil_2K	All>Human_tonsil_4K
n_top_genes	2000							
Length of sub-vectors	128				256		32	
Number of principal component	Number of principal component is based on the number of proteins measured in scProtein-seq data.							
λ	50							
Number of head	64						16	
drop	0.1						0	
epoch	50							
learning_rate	0.0001	0.0001	0.0001	0.0001	0.0001	0.001	0.001	0.001
batch_size	32							

Table S4. Datasets

	Data Name	num of cell	num of gene in scRNA-seq	num of protein in scProtein	species	tissue	Protocol	Accession ID
GEX+ADT	Mimitou_CITE_5k	4644	17441	227	Human	PBMC	CITE-seq	GSE156478
ATAC+ADT	Mimitou_ASAP_5k	4502	17441	227	Human	PBMC	ASAP-seq	GSE156479
GEX+ADT	Peterson_REAP_7k	7092	32738	43	Human	PBMC	REAP-seq	GSE100501
GEX+ADT	Seurat_CITE_50k	49147	33538	54	Human	PBMC	CITE-seq	GSE164378
GEX+ADT	NeurlPS_CITE_90k	90261	13953	134	Human	BMMC	CITE-seq	GSE194122
GEX+ADT	Seurat_CITE_160k	161764	20729	224	Human	PBMC	CITE-seq	GSE164378
GEX+ADT	Gayoso_CITE_20K	20177	28692	210	Mouse	spleen	CITE-seq	GSE150599
GEX+ADT	Gayoso_CITE_19K	19698	28692	210	Mouse	lymph nodes	CITE-seq	GSE150599
Spatial_GEX+ADT	Human_skin_2K	1691	15486	282	Human	skin	Spatial-CITE-seq	GSE213264
Spatial_GEX+ADT	Human_spleen_2K	2494	20236	282	Human	spleen	Spatial-CITE-seq	GSE213265
Spatial_GEX+ADT	Human_thymus_2K	2500	28278	282	Human	thymus	Spatial-CITE-seq	GSE213266

Spatial_GEX+ADT	Human_tonsil_2K	2492	28417	282	Human	tonsil	Spatial-CITE-seq	GSE213267
Spatial_GEX+ADT	Mouse_colon_2K	2037	19468	198	Mouse	colon	Spatial-CITE-seq	GSE213268
Spatial_GEX+ADT	Mouse_intestine_1K	902	20444	198	Mouse	intestine	Spatial-CITE-seq	GSE213269
Spatial_GEX+ADT	Mouse_kidney_2K	2419	23750	198	Mouse	kidney	Spatial-CITE-seq	GSE213270
Spatial_GEX+ADT	Mouse_spleen_1K	1303	19923	198	Mouse	spleen	Spatial-CITE-seq	GSE213271
Spatial_GEX+ADT	Human_breast_4K	4169	18085	35	Human	breast	10x Genomics Xenium	https://www.10xgenomics.com/datasets/gene-and-protein-expression-library-of-human-breast-cancer-cytassist-ffpe-2-standard
Spatial_GEX+ADT	Human_brain_6K	5756	18085	35	Human	brain	10x Genomics Xenium	https://www.10xgenomics.com/datasets/gene-and-protein-expression-library-of-human-glioblast

								oma-cytassist-ffpe-2-standard
Spatial_GEX+ADT	Human_tonsil_4K	4194	18085	35	Human	tonsil	10x Genomics Xenium	https://www.10xgenomics.com/datasets/gene-protein-expression-library-of-human-tonsil-cytassist-ffpe-2-standard
GEX+Peak+ADT	Mimitou_DOGMA_14K	13763	36495	210/68963(number of peaks)	Mouse	PBMC	DOGMA-seq	GSE156478
GEX+ADT	Stephenson_CITE_657K	647366	24,737	192	Human	PBMC	CITE-seq	https://covid19.cog.sanger.ac.uk/submissions/release2/vento_pbmc_processed.h5ad
GEX+ADT	COMBAT_CITE_836K	836148	37502	192	Human	PBMC	CITE-seq	GSE205055
Spatial_ATAC–RNA-seq	Zhang_Spatial_ATAC–RNA_2K	2187	17058	18694(number of peaks)	Mouse	Brain	Spatial_ATAC–RNA	GSE205055

Supplementary Note 1

Tools for comparison

Seurat

In this study, we employed Seurat v4. The authors introduce a computational method called "weighted-nearest neighbor" (WNN) analysis to address the challenge of integrating multiple data types for defining cellular identity. This unsupervised framework learns the relative utility of each data type in each cell and allows for an integrative analysis of multimodal data. Here,we used Harmony to perform the integrating scATAC-seq, scRNA-seq, and protein datasets.

Harmony

Harmony is an algorithm commonly utilized for the integration and harmonization of single-cell RNA sequencing (scRNA-seq) datasets obtained from multiple experimental conditions or batches. The underlying principle of Harmony involves modeling the sources of technical variability and subsequently adjusting the data to account for these variations. It employs a linear algebra framework to identify and remove the batch-specific effects, revealing the shared biological signal across different datasets. By effectively reducing the confounding effects of technical variation, Harmony enhances the biological coherence and comparability of integrated scRNA-seq datasets. Here,we used Harmony to perform the integrating scATAC-seq, scRNA-seq, and protein datasets.

LIGER

LIGER (linked inference of genomic experimental relationships) is an algorithm used for integrating multiple experimental conditions or batches of single-cell multi-omics data. Its main purpose is to fuse information from different data modalities, such as scRNA-seq and scATAC-seq, to achieve cell type consistency and enable comparative analysis across multi-omics datasets. The implementation of LIGER is based on a joint low-rank model that embeds cell features from different data modalities into a shared low-dimensional space. By minimizing the distances between different data modalities in the embedding space, LIGER accomplishes the

alignment and integration of multi-omics data. Here,we used LIGER to perform the integrating scATAC-seq, scRNA-seq.

online iNMF

Online iNMF (incremental Non-negative Matrix Factorization) is an algorithm utilized for the analysis of large-scale single-cell transcriptomic datasets. Its primary purpose is to identify biologically relevant patterns and sources of variation within the data by decomposing the gene expression matrix into non-negative factors. The online iNMF algorithm operates in an incremental manner, allowing for efficient analysis of datasets that are too large to be processed as a whole. It processes the data in batches or subsets, updating the factorization iteratively to capture the underlying structure and variability of the dataset. This incremental approach enables the algorithm to handle datasets with millions of cells and thousands of genes, making it suitable for the analysis of extensive single-cell transcriptomic data. Here,we used online iNMF to perform the integrating scATAC-seq, scRNA-seq, and protein datasets.

BindSC

BindSC is a computational method developed for the integration of single-cell multiomics profiles generated by different single-cell technologies from the same biological sample. The algorithm is based on a novel mathematical solution called bi-order canonical correlation analysis (bi-CCA), which extends the commonly used CCA approach to align the rows and columns between data matrices iteratively. Unlike existing integration methods that rely on shared features, BindSC utilizes full feature information to achieve precise alignment of cell subtypes and enables the discovery of novel gene-protein associations. Here,we used scJoint to perform the integrating scATAC-seq, scRNA-seq and protein.

scJoint

scJoint is a transfer learning method designed to integrate large-scale and heterogeneous collections of scRNA-seq and scATAC-seq data in single-cell multiomics analysis. It utilizes a semisupervised framework and neural network-based techniques to simultaneously train labeled and unlabeled data, enabling label transfer and joint visualization. The algorithm consists of three main steps: joint dimension reduction and modality alignment, label transfer via k-nearest neighbors, and

improved mixing between modalities using **metric learning**. Here,we used scJoint to perform the integrating scATAC-seq, scRNA-seq.

Pamona

Pamona is an algorithm designed for the integration of heterogeneous single-cell multi-omics sequencing data. It addresses the challenge of aligning and representing shared and dataset-specific cellular structures across different modalities. The algorithm formulates this task as a partial manifold alignment problem and utilizes a partial Gromov-Wasserstein optimal transport framework to solve it. Pamona identifies shared and dataset-specific cells based on probabilistic couplings and aligns the cellular modalities in a common low-dimensional space while preserving both shared and dataset-specific structures. It can incorporate prior information such as cell type annotations or cell-cell correspondence to improve alignment quality. Here,we used Pamona to perform the integrating scATAC-seq, scRNA-seq and protein

UnionCom

UnionCom is an algorithm developed for the unsupervised topological alignment of single-cell multi-omics data integration. It addresses the challenge of integrating datasets consisting of unpaired cells measured with distinct unmatched features across modalities. The algorithm works by first embedding the intrinsic low-dimensional structure of each single-cell dataset into a distance matrix of cells within the same dataset. Then, it aligns the cells across datasets by matching the distance matrices through a matrix optimization method. Finally, UnionCom projects the distinct unmatched features into a common embedding space for feature comparability of the aligned cells. The key advantages of UnionCom are its unsupervised and data-driven nature, its ability to handle non-linear intrinsic structures, and its capacity to accommodate samples with dataset-specific cell types. It does not require correspondence information among cells or features, making it suitable for integrating single-cell multi-omics datasets. Here,we used UnionCom to perform the integrating scATAC-seq, scRNA-seq and protein

scVI

scVI (single-cell variational inference) is a scalable framework for the probabilistic representation and analysis of gene expression in single cells. It

addresses the challenges of technical noise and bias in single-cell transcriptome measurements, providing a ready-to-use solution for downstream analyses. The main purpose of scVI is to model and account for the uncertainty in gene expression data at the single-cell level. It utilizes stochastic optimization and deep neural networks to aggregate information across similar cells and genes, while considering batch effects and limited sensitivity. By approximating the underlying distributions of observed expression values, scVI enables various analysis tasks such as batch correction, visualization, clustering, and differential expression. The algorithm models the observed gene expression of each cell as a sample drawn from a zero-inflated negative binomial (ZINB) distribution. It incorporates additional random variables to capture nuisance variation and biological differences between cells. A neural network is employed to map the latent variables to the parameters of the ZINB distribution, enabling efficient analysis and imputation of missing values. Here,we used scVI to perform the integrating scATAC-seq, scRNA-seq and protein

TotalVI

TotalVI is a framework designed for the joint analysis of paired RNA and protein measurements in single cells using the cellular indexing of transcriptomes and epitopes by sequencing (CITE-seq) technique. Its main purpose is to integrate these paired views into a unified representation of cell states, overcoming the technical challenges associated with each measurement. The totalVI algorithm employs total variational inference, a probabilistic latent variable model, to capture the uncertainty in observed RNA and protein counts. It represents the data as a composite of biological and technical factors, accounting for sources of variation such as protein background and batch effects. By optimizing the parameters of its components using the variational autoencoder (VAE) framework, totalVI achieves efficient and scalable analysis of CITE-seq data.

BABEL

BABEL is a deep learning algorithm that addresses the challenge of simultaneous profiling of multiple modalities within a single cell in the field of single-cell biology. Its main purpose is to translate between chromatin, RNA, and protein profiles of

single cells, allowing the computational synthesis of matched multiomic measurements when only one modality is experimentally available. The algorithm consists of four modular neural networks: two encoders and two decoders. The encoders project either RNA or ATAC profiles (chromatin accessibility) into a shared latent representation, while the decoders infer their corresponding profiles from this latent representation. This shared latent space serves as an abstract, integrated representation of cellular state, capturing major cellular variations. BABEL is trained using a loss function that requires both encoders to be interoperable with either decoder, enabling the translation across different modalities. It leverages paired data to learn a unified latent space without explicit alignment methods. The model predicts gene expression from chromatin accessibility and vice versa, using negative binomial and binary cross entropy loss functions, respectively.

sciPENN

sciPENN is a versatile deep learning algorithm designed to address the challenges of integrating and analyzing CITE-seq and scRNA-seq data in single-cell multi-omics studies. Its main purpose is to support data integration, protein expression prediction, protein expression imputation, uncertainty quantification, and cell type label transfer. Its implementation involves a network structure comprising various layers and blocks, along with a censored loss function scheme.

scMM

scMM (single-cell Mixture-of-Experts Multiomics) is a deep generative model designed for integrated analysis of single-cell multiomics data. It addresses the challenge of analyzing complex and high-dimensional multimodal single-cell data by inferring interpretable joint representations and enabling crossmodal generation of single-cell data. The algorithmic implementation of scMM involves a mixture-of-experts framework, consisting of four neural networks with an encoder-decoder pair for each modality. The encoders are used to infer the variational posterior, from which latent variables are sampled. The decoders calculate the parameters of probability distributions (such as negative binomial or zero-inflated negative binomial) to model the characteristics of each modality's data. scMM is

extensible and can be applied to various modalities by constructing the model with different distributions. It has potential applications in other multimodal data types, such as single-cell transcriptome and DNA methylome.

Supplementary Method 1. scmFormer for integrating unpaired single-cell multi-omics data

In numerous instances, single-cell multi-omics datasets exhibit inherent imbalance or lack of pairing, stemming from diverse factors such as irregular sampling practices and technical limitations. This unpaired nature of the datasets presents a significant analytical challenge, necessitating robust integration methods to address the disconnect. To tackle this issue, we have advanced the capabilities of scmFormer to effectively process unpaired data.

Unlike the original scmFormer, this iteration of scmFormer is tailored to classify different modalities, employing solely the data recovery loss for optimization. More specifically, let us consider two distinct modalities of data obtained: X_{mod1} and X_{mod2}, where the cells within each modality are not matched. scmFormer, initially trained on the first modality X_{mod1}, is preserved; subsequently, training continues on the second modality X_{mod2}, leveraging the pre-trained scmFormer. This approach ensures that the latent representation of the second modality progressively aligns with that of the first. Through this iterative training process, we obtain congruent latent representations for both modalities. After securing these latent representations, we apply linear optimal transport (Supplementary Method 2) to map the latent space of X_{mod1} onto that of X_{mod2}, thereby achieving integration of the unpaired datasets.

Supplementary Method 2. Linear optimal transport

Our objective is to find an optimal linear mapping when dealing with data from two single-cell datasets, making their distributions as similar as possible. This mapping considers factors such as mean, weights, and covariance of single-cell data. By minimizing the distribution differences between the source and target datasets, we aim to enhance their comparability and integrability.

Our approach is grounded in optimal transport theory, minimizing the

transportation cost between two single-cell datasets to find an optimal linear mapping. This mapping incorporates statistical features from both the source and target datasets, ensuring the preservation of crucial information during the integration process.

Method Steps:

Data Input:

Source dataset X_s: $Xs \in \mathrm{R}^{m \times d}$ where m is the number of cell and d is the dimensionality of the features.

Target dataset X_t: $X_t \in \mathrm{R}^{n \times d}$ where n is the number of cell and d is the dimensionality of the features.

Estimation of Distribution Parameters:

Estimate the mean vectors μ^s for the source and μ^t for the target dataset:

$$\mu_s = \frac{1}{m}\sum_{i=1}^{m} X_{s_i}, \mu_t = \frac{1}{n}\sum_{i=1}^{n} X_{t_i} . \tag{1}$$

The mean vectors provide a central reference for each dataset, which is instrumental in aligning the datasets in subsequent steps. The mean calculation informs us about the central tendency of the datasets.

Centering the Datasets:

This involves subtracting the mean vector from each dataset (if bias is considered in the model):

The datasets are centered by subtracting their respective mean vectors:

$$\tilde{X}_s = X_s - \mathbf{1}\mu_s^T, \tilde{X}_t = X_t - \mathbf{1}\mu_t^T , \tag{2}$$

Where **1** is a column vector of ones.

Centering the data removes the mean offset between the two distributions, making them zero-mean. This simplifies the computation of the covariance matrices and ensures that the subsequent transport plan focuses on the shape of the distributions rather than their location.

Covariance Matrix Calculation:

Calculate the weighted covariance matrices C_s for the source and C_t for the target dataset:

$$C_s = \frac{1}{m-1} \tilde{X}_s^T \tilde{X}_s, C_t = \frac{1}{m-1} \tilde{X}_t^T \tilde{X}_t. \quad (3)$$

Covariance matrices capture the spread and the shape of the data distributions. By computing these, we understand how variables within each dataset vary together.

Calculation of the Mapping Matrix:

Using the target covariance matrix C_t and the source covariance matrix's square root $C_s^{1/2}$ compute the mapping matrix A:

$$M_0 = C_s^{1/2} C_t C_s^{1/2}, \quad (4)$$

$$A = C_s^{-1/2} M C_s^{-1/2}. \quad (5)$$

The mapping matrix A is a linear transformation that reshapes the source covariance matrix to match the target covariance matrix as closely as possible. The square roots and inverse square roots of the covariance matrix are computed to normalize and then denormalize the data into the space of the target distribution.

Calculation of the Bias Vector:

Apply the mapping matrix A and bias vector b to the source dataset:

$$X_1 = X_s A + \mathbf{1}\mathrm{b}^{\mathrm{T}}. \quad (6)$$

By applying the optimal transport mapping, we transport the source data to a space where its distribution is aligned with that of the target. This is the core of the method and allows for the analysis of the source data as if it were sampled from the target distribution.

The benefit of this method is that it provides a principled approach to aligning two distributions without the need for complex non-linear transformations. It's particularly advantageous when you have labeled data in one domain (source) and want to apply a model to a different but related domain (target) where labels might be scarce or expensive to obtain. This method enables better model transferability and generalization across different domains.

Supplementary Method 3. scmFormer for generation unmeasured data

scmFormer is Transformer-based models. The Transformer is a deep learning model that was introduced by Vaswani et al. in their groundbreaking 2017 paper "Attention Is All You Need," outperforming other models in tasks such as language translation. The intrinsic connections among different single-cell omics, such as transcriptomics, reveal patterns of gene activity at specific moments at the single-cell level, i.e., the expression of RNA. Proteomics, in turn, provides insights into the abundance and state of proteins translated from these RNA molecules. These two are intimately linked, as protein production and function are directly contingent upon RNA expression patterns. Yet, this relationship is not linear, being modulated by factors such as post-transcriptional regulation, translation efficiency, and protein degradation. In single-cell multi-omics, one modality of omics data can be likened to a language, and the process of inferring another omics data type from one could be analogous to translating between different languages.

Building on these assumptions and foundational theories, we posit the following hypothesis: A pre-trained scmFormer may have the potential to reveal biological principles shared across different tissues or analytical technologies, akin to a "universal grammar" in linguistic theory. Drawing an analogy to natural language processing (NLP), if we treat gene expression profiles as complex structured data points or 'sentences', NLP techniques could be utilized to decode patterns and predict unmeasured modalities. This hypothesis suggests that applying pre-trained models to novel tissues and technologies could be a fruitful avenue for exploration, offering a versatile approach to mining biological insights from intricate datasets.

We have expanded the scope of scmFormer to include the generation of unmeasured modalities. Specifically, we modified the model's input structure: during training, for the modality we aimed to predict, we set its corresponding input information to zero (X_{zero}), while retaining the information for the other modality X_{input} = (X_{mod1}, X_{zero}). The model thus outputs both Modality 1 and Modality 2 information,

X_{output} = ($X_{reconstruction\_\mathrm{mod1}}$, $X_{prediction\_\mathrm{mod2}}$). For this task, the scmFormer model employs a Mean Squared Error (MSE) loss function for its training process. This loss function is defined as MSE(X_{output}, Y), where Y = (X_{mod1}, X_{mod2}) represents the true values. This MSE loss helps in quantifying the discrepancy between the model's predictions and the actual observed data, guiding the model to improve its accuracy in predicting unknown modalities.

In practice, the success of utilizing pre-trained models for this purpose is contingent upon the availability of extensive paired datasets, which are currently limited. In future work, we plan to actively collect more paired single-cell multi-omics data and explore strategies to train a more robust generator for unknown single-cell modalities with limited paired datasets.
